# Exploring the Capability of Mechanically Interlocked Molecules in Anion Recognition: A Computational Insight

**DOI:** 10.1021/acsphyschemau.4c00089

**Published:** 2024-12-10

**Authors:** Fábio J. Amorim, Giovanni F. Caramori

**Affiliations:** Departamento de Química, Universidade Federal de Santa Catarina, Campus Universitário Trindade, 88040-900 Florianópolis, SC, Brazil

**Keywords:** mechanically interlocked molecules, GKS-EDA, DFT, anion recognition, hydrogen bond, halogen bond, chalcogen bond

## Abstract

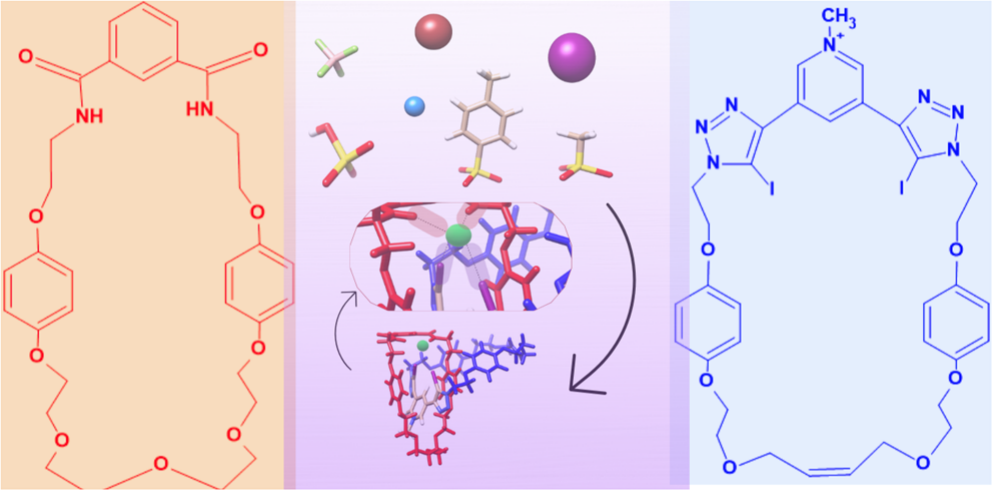

The present study elucidated the role of both hydrogen and halogen bonds, from an electronic structure perspective, in the anion recognition process by the [2]catenane (**1**) containing a moiety with hydrogen bond donors entangled with another macrocyclic halogen bond donor. Spherical and nonspherical anions have been employed. The roles of different σ–hole donors have also been considered. The structure of **1** was modified by incorporating other σ–hole donors, namely bromine, chlorine, fluorine, as well as −Te–CH_3_ as a chalcogen bond donor, leading to the modified [2]catenanes **2**–**5**. Insights into anion recognition were gained by quantifying the contributions of not only the mechanical but also hydrogen and halogen/chalcogen bonds to anion recognition using the GKS-EDA energy partition scheme and homodesmostic reactions scheme. GKS-EDA reveals that the anions Cl^–^ and TS^–^ exhibit the most stabilizing interactions with the **1** binding pocket. The EDA results confirm that by changing from a stronger σ-hole donor (I) to a weaker σ-hole donor (F) will have a considerable impact on anion interaction, thereby demonstrating that the halogen bonds formed between the [2]catenane and the anion play a pivotal role.

## 1 Introduction

The study of mechanical bonds (MB) is of paramount importance in supramolecular chemistry. It is a fundamental aspect of the development of molecular-level machines involved in mechanochemical processes.^1^ A mechanical bond is defined as an equilibrium-based interplay between attractive and repulsive forces resulting from the entanglement of two or more component fragments, forming a structure that cannot be disentangled without breaking regular chemical bonds between its atoms. Molecules that contain such bonds are referred to as mechanically interlocked molecules (MIMs).^2,3^

**Figure 1:**
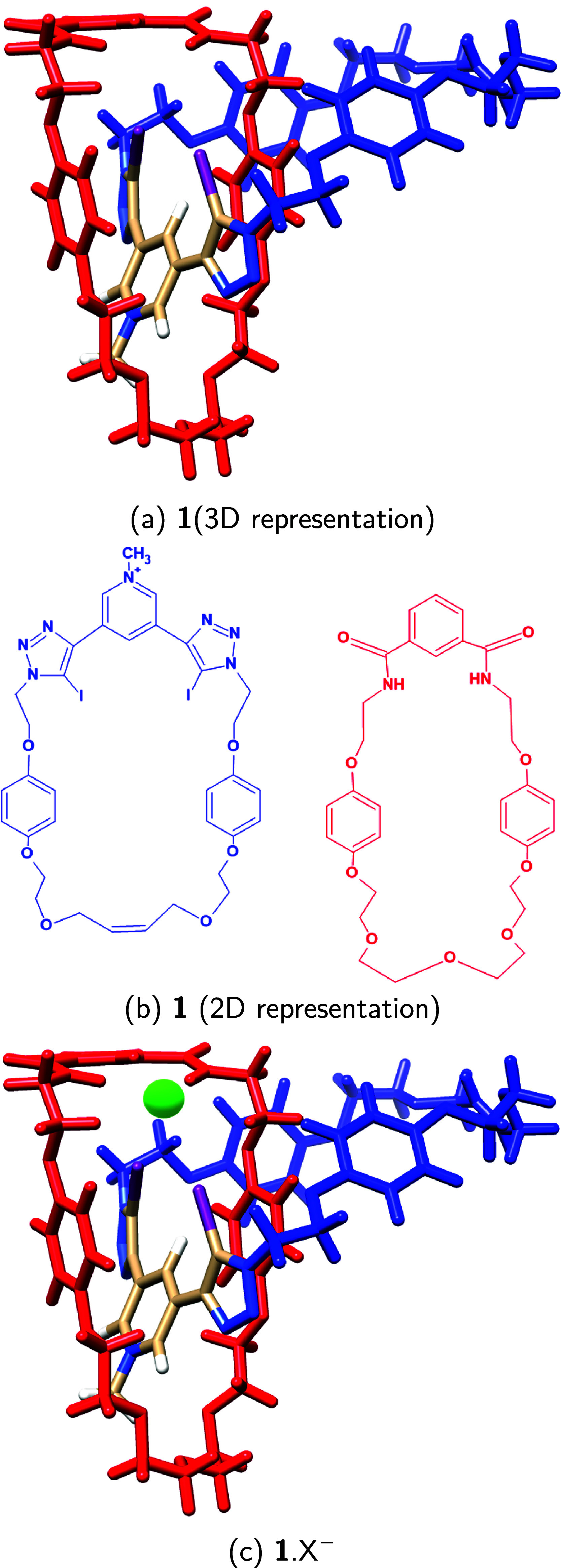
(a) 3D representation of [2]catenane (**1**) containing a bis-iodine-triazole moiety (highlighted). (b) Chemical structures of its constituents in 2D representation. (c) Schematic representation of **1**.X^–^ showing the cleft in which recognition of the anion (represented by the green sphere) takes place.

Catenanes, in conjunction with rotaxanes and molecular knots, constitute a representative class of MIMs.^4^ Catenanes comprise a chain-like structure formed by the linkage of two (or more) ring-shaped molecules, as illustrated in Figure 1.^5^ In the context of mechanically interlocked molecules, it is reasonable to consider the potential of catenanes for the development of ion recognition systems by exploiting the structural and chemical properties inherent to this class of MIMs.^6^ This process enables the formation of effective structures that facilitate the binding of charged species, thereby providing the essential chemical input required for the activation of the molecular machines.^7^ In the case of anions, although this area of research has been a focus of attention in supramolecular chemistry,^8,9^ it has also presented a challenge due to the intrinsic properties of anions.

In the realm of molecular recognition, molecules capable of binding cations and/or anions typically engage in a diverse array of noncovalent interactions either alone or in combination with each other, such as hydrogen bonds,^10−14^ electrostatic interactions,^15,16^ metal-ion coordinations,^17^ anion−π interactions,^18^ halogen bonds,^19,20^ and interactions driven by hydrophilicity.^21,22^ Mechanically interlocked molecules (MIMs) have attracted attention as promising receptors for ionic species recognition. The interlocked architecture of their structure provides well-defined cavities and clefts that offer enhanced and potentially more favorable noncovalent interactions, making them more prone to interact with ionic species. This feature demonstrates the potential of MIMs as effective and selective hosts for cations and anions in a range of chemical and biological applications.^23,24^ In the context of anion recognition, the potential of halogen bonds as a means of enhancing selectivity has been investigated. The primary interaction between the MIM structure containing the halogen bond donor atom(s) and the negatively charged species comes from the σ–hole interaction.^9,25^ It has been previously demonstrated that such interactions are more suited to anion recognition than the utilization of hydrogen bonding (H-bond).^26,27^

**Figure 2:**
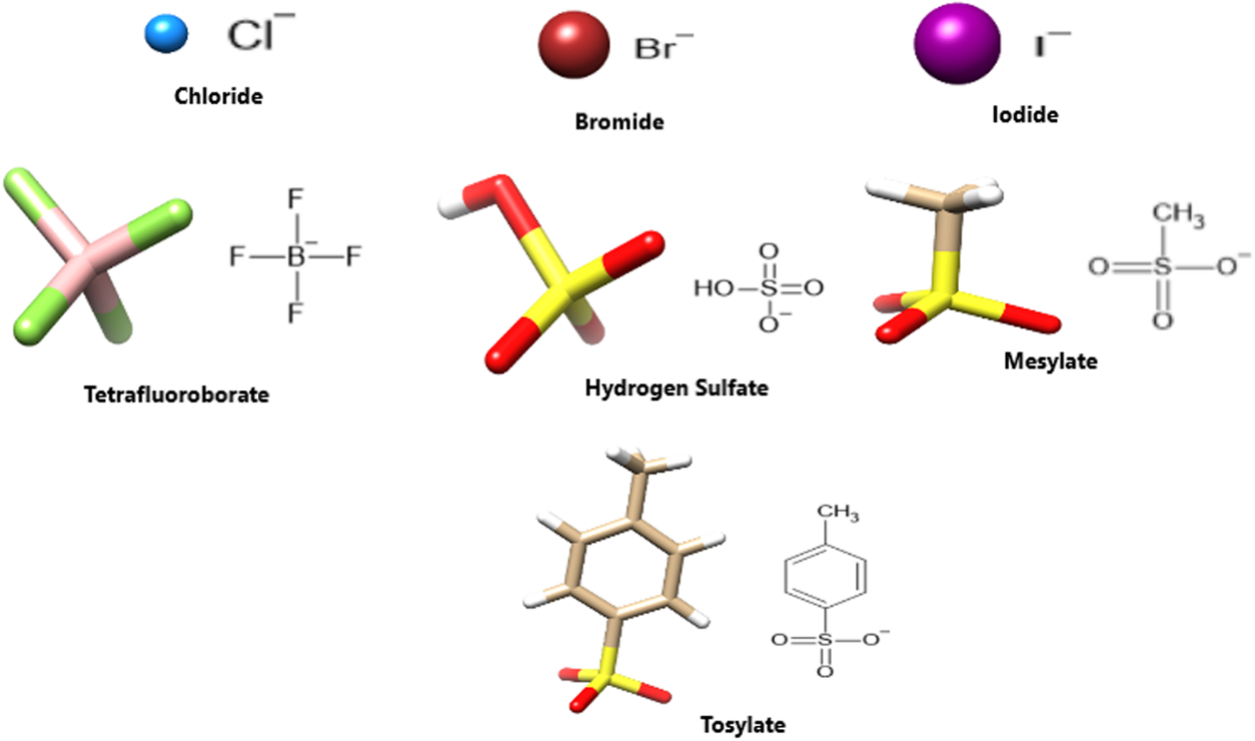
Anions of charge −1 considered in the present study, including the spherical halides: Cl^–^, Br^–^, I^–^; and the nonspherical anions: BF_4_^–^, HSO_4_^–^, MS^–^, and TS^–^.

The present study elucidates the role of both hydrogen and halogen bonds, from an electronic structure perspective, in the anion recognition process by the [2]catenane (**1**) under consideration,^27^ which contains a macrocycle (shown in red) with two hydrogen bond donors entangled with another macrocyclic component (shown in blue) containing the bis-iodo-triazole pyridinium (highlighted) as a halogen bond donor (Figure 1). For recognition, a set of anions with charge −1 were considered, including the spherical halide ions (Cl^–^, Br^–^, and I^–^) as well as nonspherical anions (BF_4_^–^, HSO_4_^–^, mesylate MS^–^, and tosylate TS^–^), as depicted in Figure 2.

In order to gain further insight into the role of σ–hole interactions, particularly on their strength, different σ–hole donors have been considered. In this way, the structure of **1** was modified by incorporating other sigma hole donors, namely bromine, chlorine, and fluorine. Furthermore, a substitution with tellurium (−Te–CH_3_) as a chalcogen bond donor was employed, leading to the modified [2]catenanes **2**–**5** (Figure S1, Supporting Information material). The effect of counterion was also taken into account by considering PF_6_^–^ as counterion. Generalized Kohn–Sham energy decomposition analysis, GKS-EDA, has been used as a quantitative tool in various fragmentation schemes to provide a deeper insight into the role that mechanical bonds as well as hydrogen, halogen, and chalcogen bonds may play in anion recognition.

## 2 Computational Methods

The crystallographic structure of the [2]catenane **1** was employed as a reference model^27^ to build up the structures **2**–**5**, in which the halogen bond donor has been changed as shown in Figure S1. The anions (Figure 2) have been positioned in the cleft formed between the moieties containing hydrogen bonding and halogen/chalcogen bonding donor groups, as this configuration has been shown to be optimal based on experimental data.^27^ Subsequently, the entire structure ([2]catenane + anion) was fully optimized without constraints using the generalized gradient approximation (GGA) density functional theory (DFT) functional of Becke^28^ and Perdew,^29^ BP86, in conjunction with the application of Grimme’s dispersion correction, DFT-D4,^30^ and the basis set LANL2DZ.^31^ Auxiliary basis sets were also employed following the resolution of the identity approximation (RI). It has been considered for both the Coulomb (def2/J)^32^ and the exchange-correlation integrals (RIJCOSX).^33^ All of the above structures were fully optimized without constraints using the same level of theory that was used to optimize **1**. All optimized geometries correspond to minima structures on the potential energy surface due to the absence of imaginary eigenvalues on the Hessian matrix. All geometry optimizations were performed using the software Orca5.04^34^ without constraints and considering implicit solvation with SMD^35^ model and dichloromethane as solvent. The molecular graphics images were created using the UCSF Chimera package.^36^

Further insight into anion recognition was gained by quantifying the contributions of not only the mechanical but also hydrogen and halogen/chalcogen bonds to anion recognition using a specific energy partition scheme, called GKS-EDA, which allows for the quantification of the contributions of these bonds to anion recognition. The influence of counterions on the GKS-EDA components was taken into account by considering the presence of the PF_6_^–^ in the detection of the chloride ion (**6**). The nature of the host–guest interaction between the MIMs and the chosen anions was analyzed with the usage of the general Kohm–Sham energy decomposition analysis (GKS-EDA)^37,38^ approach as implemented in GAMESS-US software,^39^ available at the Xiamen Atomistic Computing Suite. The present analysis was conducted using the same theoretical framework previously outlined but now applied with a valence-only basis set, namely SBKJC-ECP.^40,41^ To support the reasoning behind the GKS-EDA results, Hirshfeld charge analysis was also considered^42^ utilizing the same level of theory and dispersion correction previously mentioned but in conjunction with the def2-SVP^43^ basis set. The GKS-EDA has been employed focusing to shed light on the bonding situations involving the ability of [2]catenanes **1**–**6** to recognize anions, as well as to quantify the contributions of mechanical bonds and hydrogen, halogen, and chalcogen bonds on this process, by means of different partition schemes adopted. In this method, the total interaction energy, Δ*E*^tot^, between two or more interacting fragments is decomposed according to eq 1, in which the total energy is the difference between the energy of the whole structure under consideration, commonly called “supermolecule,” *E*_S_ and the sum of fragment energy *E_f_*. Δ*E*^tot^ is decomposed into physically meaningful terms: electrostatic (Δ*E*^elstat^), exchange (Δ*E*^ex^), repulsion (Δ*E*^rep^), polarization (Δ*E*^pol^), electron correlation (Δ*E*^corr^), and dispersion (Δ*E*^disp^).
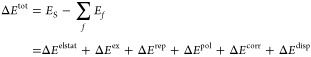
1

The stabilizing components (polarization, dispersion, and correlation) can be grouped, resulting in the so-called orbital term Δ*E*^oi^, in canonical energy decomposition analysis (eq 2). Similarly, exchange and repulsion are typically grouped together under the designation of Pauli’s repulsion term, Δ*E*^pauli^ (eq 3).^38^
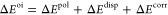
2
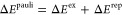
3

## 3 Results and Discussion

**Figure 3:**
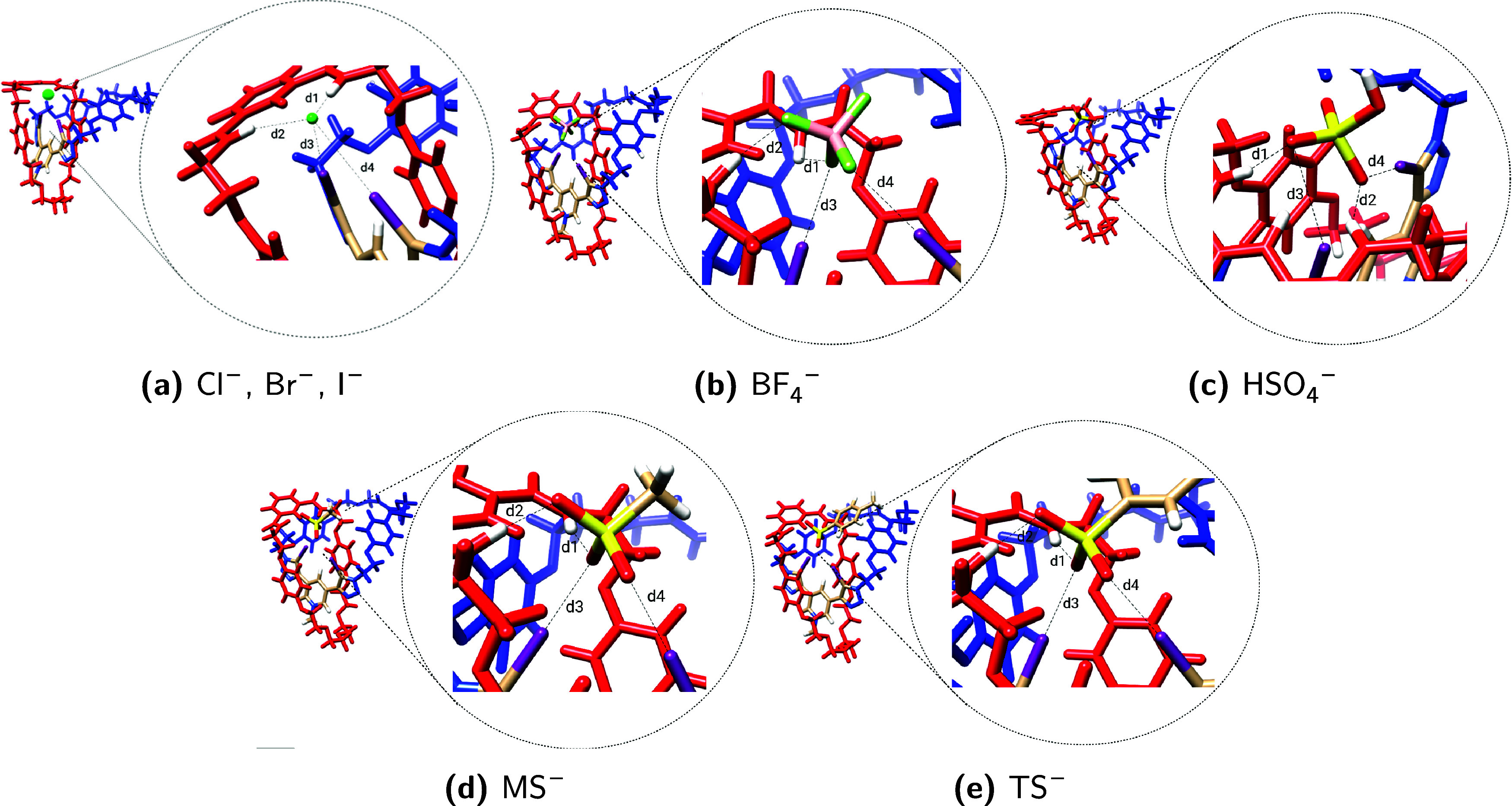
Geometrical parameters involved in the recognition of anions depicted in Figure 2 and [2]catenanes **1**–**6**. The distances **d1** and **d2** are the shortest distances between the hydrogen bond donor and acceptor, while **d3** and **d4** depict the shortest distances between the σ-hole donor and acceptor (see insets).

In all cases, after geometry optimization, the anions converged to a position inside the cleft formed by the two [2]catenane moieties, where they are stabilized by both hydrogen bonds and halogen or chalcogen bonds due to the presence of σ–hole donors. Table 1 provides a concise overview of the most significant geometrical parameters associated with anion interaction. **d1** and **d2** depict the shortest distances between the hydrogen bond donor and acceptor, while **d3** and **d4** are the shortest distances between the σ–hole donor and acceptor (See insets of Figure 3). The hydrogen bond angles of N–H_1_···X^–^ and N–H_2_···X^–^ are also reported.

**Table 1:** Selected Geometric Parameters for the Optimized Structures of **1**.X^–^ (X = Cl, Br, I, BF_4_, HSO_4_, MS, and TS), **2**–**5**.X^–^ (X = Cl, C7H7SO3), and **6**.Cl^–^ (Counterion PF_6_^–^)^a^

	angles (deg)		distances (Å)				stretches (cm^–^)	
	N–H_1_···X^–^	N–H_2_···X^–^	**d1**	**d2**	**d3**	**d4**	N–H_1_	N–H_2_
**1**	–	–	–	–	–	–	3542.40	3482.09
**1**.Cl^–^	167.2	166.8	2.36	2.31	3.20	3.19	3336.12	3322.27
	(165.1)^b^	(164.8)	(2.63)	(2.61)	(3.30)	(3.11)		
**1**.Br^–^	163.7	163.5	2.55	2.52	3.36	3.35	3368.89	3358.66
	(148.0)	(161.1)	(2.82)	(2.76)	(3.36)	(3.18)		
**1**.I^–^	157.2	158.9	2.84	2.83	3.57	3.59	3420.15	3417.02
	(149.7)	(146.9)	(3.01)	(3.02)	(3.54)	(3.32)		
**1**.BF_4_^–^	110.5	165.1	2.72	1.92	2.87	2.93	3507.04	3426.62
**1**.HSO_4_^–^	166.2	148.7	1.78	2.10	4.47	2.97	3430.02	3269.34
**1**.MS^–^	162.7	165.8	1.80	1.76	2.71	2.74	3228.08	3150.45
**1**.TS^–^	164.3	167.1	1.80	1.79	2.70	2.73	3230.44	3172.50
**2**.Cl^–^	166.8	166.8	2.33	2.32	3.06	3.19	3313.48	3295.27
**2.**TS^–^	164.4	167.3	1.80	1.80	2.87	2.91	3218.09	3166.21
**3**.Cl^–^	167.3	168.1	2.28	2.30	3.16	3.34	3262.41	3232.43
**3**.TS^–^	164.3	169.2	1.77	1.74	2.76	2.79	3172.50	3059.80
**4**.Cl^–^	166.5	166.0	2.26	2.25	3.77	4.13	3213.96	3178.35
**4**.TS^–^	164.9	171.2	1.75	1.71	3.03	3.05	3120.38	3014.69
**5**.Cl^–^	166.8	166.3	2.34	2.31	3.70	3.15	3305.60	3286.75
**5**.TS^–^	168.1	167.7	1.77	1.74	2.71	2.75	3196.27	3127.52
**6**.Cl^–^	167.3	166.4	2.36	2.31	3.19	3.20	3334.03	3319.19

a Stretching frequencies of hydrogen bond donors, N–H_1_ and N–H_2_, are reported in units of cm^–1^.

b Crystallographic data available in ref (27).

The values reported in Table 1 confirm that there are both σ–hole and hydrogen bond interactions happening between the anions and **1** given the favorable **d3** and **d4** distances for the σ–hole interaction, as well as the almost linear N–H_1_···X^–^ and N–H_2_···X^–^ angles and the hydrogen bond distances **d1** and **d2**. Another evidence supporting the stabilization of the anions by the hydrogen bonds is the red shift in the stretching frequencies of the N–H_1_ and N–H_2_ bonds when they start to interact with the anions, confirming that the anions X^–^ attract the H to themselves, lengthening the NH_1_ and NH_2_ bond lengths and causing the red shifts, which occur to a greater or lesser degree, depending on both the minimum conformation adopted by the [2]catenane and the nature of the interacting anion (Table 1).

Moreover, the spherical halide ions (Cl^–^, Br^–^, and I^–^) interact favorably with the hydrogen bond and σ hole donors of **1**. The distances **d1** and **d2** are slightly smaller than those for **d3** and **d4**, but consistent with each other, increasing with anion radius. For example, **d1** and **d2** vary from 2.31 to 2.84 Å when going from Cl^–^ to I^–^, while **d3** and **d4** vary from 3.19 to 3.59 Å. Furthermore, the angles N–H_1_···X^–^ and N–H_2_···X^–^ become more bent on going from Cl^–^ to I^–^, suggesting that the interactions become less stabilizing as the size of the halide ion increases. Indeed, these geometric parameters are entirely consistent with the energy decomposition analysis, GKS-EDA (Table 2), which corroborates that the chloride ion establishes the most favorable interaction with **1**, while the iodide anion, with the largest atomic radius, will likely have the least favorable values of interaction. The calculated geometric parameters reproduce the trends observed in the crystallographic data available for **1**.Cl^–^,**1**.Br^–^, and **1**.I^–^, supporting the efficacy of the employed level of theory in elucidating the observed experimental trends.

**Table 2:** GKS-EDA Results Obtained through the Interaction Between Two Fragments Where the First Fragment is the Studied [2]Catenane and the Second Fragment Being the Applied Anions

										**HPS**	
	Δ*E*^tot^	Δ*E*^elstat^	Δ*E*^ex^	Δ*E*^rep^	Δ*E*^pol^	Δ*E*^corr^	Δ*E*^disp^	Δ*E*^pauli^^b^	Δ*E*^oi^^c^	catenane	anion
**1.Cl**^–^	–115.1	–107.4	–121.5	186.9	–50.3	–15.8	–7.0	65.4	–73.0	0.38	–0.38
		(35.6%)^a^	(40.2%)		(16.7%)	(5.2%)	(2.3%)				
**1.Br**^–^	–110.6	–101.6	–110.0	170.1	–43.7	–17.2	–8.2	60.1	–69.1	0.39	–0.39
		(36.2%)	(39.2%)		(15.6%)	(6.1%)	(2.9%)				
**1.I**^–^	–104.8	–91.9	–95.8	149.2	–39.0	–18.0	–9.3	53.4	–66.3	0.47	–0.47
		(36.2%)	(37.7%)		(15.3%)	(7.1%)	(3.7%)				
**1.BF**_**4**_^–^	–87.6	–79.3	–51.7	80.0	–19.8	–5.7	–11.1	28.3	–36.6	0.66	– 0.66
		(47.3%)	(30.8%)		(11.8%)	(3.4%)	(6.6%)				
**1.HSO**_**4**_^–^	–104.2	–100.9	–100.3	161.5	–38.6	–10.0	–15.8	61.2	–64.4	0.42	–0.42
		(38.0%)	(37.8%)		(14.5%)	(3.8%)	(5.9%)				
**1.MS**^–^	–116.4	–118.0	–136.9	223.5	–57.7	–10.4	–17.0	86.6	–85.1	0.27	–0.27
		(34.7%)	(40.2%)		(17.0%)	(3.1%)	(5.0%)				
**1.TS**^–^	–123.4	–117.9	–148.0	241.7	–62.7	–9.9	– 26.7	93.7	–99.3	0.26	–0.26
		(32.3%)	(40.5%)		(17.2%)	(2.7%)	(7.3%)				

a % of attractive int. (Δ*E*^elstat^ + Δ*E*^ex^ + Δ*E*^pol^ + Δ*E*^corr^ + Δ*E*^disp^).

b = Δ*E*^ex^ + Δ*E*^rep^.

c = Δ*E*^pol^ + Δ*E*^disp^ + Δ*E*^corr^.

For the observed geometric parameters in **6**.Cl^–^, it is noteworthy that the obtained results reported in Table 1 are almost identical to the behavior in **1**.Cl^–^, including both N–H stretches. This behavior is to be expected, given that the only difference between the two is the inclusion of PF_6_^–^ as a counterion. This indicates that the main difference is observed in the interaction between the anion and [2]catenane, as confirmed by the data in Table 4.

In the case of interactions involving nonspherical anions (BF_4_^–^, HSO_4_^–^, MS^–^, and TS^–^) with **1**, (Table 1), it can be observed that, given the greater availability of hydrogen bond receptors in comparison to spherical anions, there is a greater tendency for the formation of hydrogen bonds. This does not necessarily imply that the primary interaction between the nonspherical anions and **1** is exclusively through hydrogen bonds. However, this suggests that the formed hydrogen bonds may play a more substantial role in the overall stability of the system when compared to the spherical anions. It is noteworthy that some nonspherical anions, such as BF_4_^–^ and HSO_4_^–^, exhibit reduced favorable hydrogen bond angles in one of their receptors and extended distances relative to halogen bond donors (iodine atoms). As evidenced by the **d3** and **d4** values, it can be reasonably inferred that these anions will have lesser values of interaction with the [2]catenane under study. Conversely, the more favorable distances and angles within the active site of **1** suggest that the interaction energies of the TS^–^ and MS^–^ anions will be more favorable. Indeed, not only GKS-EDA (Table 2) but also the red shifts in the stretching frequencies of NH_1_ and NH_2_ corroborate this claim, demonstrating that MS^–^ and TS^–^ exhibit the most stabilizing interactions with **1**, −116.4, and −123.4 kcal mol^–1^, respectively.

As the most stabilizing interaction energies with **1** were presented by Cl^–^ and TS^–^, these anions were selected for further investigation into the effects stemming from the σ–hole donor nature. Their interactions with [2]catenanes **2**–**5** were thus studied and are described as follows. As illustrated in Table 1, regardless of the nature of the σ-hole donor employed (**2**—bromine; **3**—chlorine; **4**—fluorine; and **5**—tellurium (−Te–CH_3_)), a comparable pattern in the geometrical parameters can be observed. For instance, concerning the hydrogen bond angles N–H_1_···X^–^ and N–H_2_···X^–^, while chloride interacts forming almost linear bond angles with values ranging from 166.0 to 168.1°, the tosylate anion establishes hydrogen bonds with less symmetrical bond angles, being N–H_1_···X^–^more bent, with values ranging from 164.3 to 168.1°. In contrast, N–H_2_···X^–^ is more linear, with bond angles ranging from 167.3 to 171.2°. This finding is in accordance with the observed values for the **d1** and **d2** bond distances, which are consistently shorter for the tosylate anion (1.71–1.80 Å) than for the chloride anion (2.25–2.34 Å). Moreover, halogen bond interactions appear to be more pronounced in the context of TS^–^ than Cl^–^ recognition, with the former exhibiting **d3** and **d4** values spanning from 2.71 to 3.05 Å, whereas for the latter, these values range from 3.06 to 4.13 Å. The aforementioned claims concerning geometrical parameters are corroborated by the values reported in Tables 2 and 3, which indicate that the total interaction energy values are consistently more stabilizing for the tosylate than those for the chloride anion. The details concerning the GKS-EDA outcomes are discussed in the subsequent section.

**Table 3:** GKS-EDA Results (kcal.mol^–1^) for Two Different Fragmentation Schemes

										Hirshfeld population analysis	
	Δ*E*^tot^	Δ*E*^elstat^	Δ*E*^ex^	Δ*E*^rep^	Δ*E*^pol^	Δ*E*^corr^	Δ*E*^disp^	Δ*E*^pauli^^b^	Δ*E*^oi^^c^^c^	[2]catenane	Anion
Two-body Fragmentation											
**n**.Cl^–^											
**1**	–115.1	–107.4	–121.5	186.9	–50.3	–15.8	–7.0	65.4	–73.0	0.38	–0.38
		(35.6%)^a^	(40.2%)		(16.7%)	(5.2%)	(2.3%)				
**2**	–111.3	–108.1	–112.2	173.5	–39.7	–17.7	–7.2	61.3	–64.6	0.43	–0.43
		(37,9%)	(39.4%)		(14.0%)	(6.2%)	(2.5%)				
**3**	–104.0	–99.8	–98.4	152.3	–34.2	–16.5	–7.5	53.9	–58.2	0.50	–0.50
		(38.9%)^a^	(38.4%)		(13.4%)	(6.4%)	(2.9%)				
**4**	–95.1	–92.6	–78.4	122.5	–27.6	–12.2	–6.8	44.1	–46.6	0.56	–0.56
		(42.6%)	(36.0%)		(12.7%)	(5.6%)	(3.1%)				
**5**	–112.3	–105.5	–116.2	176.9	–46.9	–12.5	–8.1	60.7	–67.5	0.42	–0.42
		(36.5%)	(40.2%)		(16.2%)	(4.3%)	(2.8%)				
**n**.TS^–^											
**1**	–123.4	–117.9	–148.0	242.3	–62.7	–9.9	– 26.7	94.3	–99.3	0.26	–0.26
		(32.2%)	(40.5%)		(17.2%)	(2.7%)	(7.3%)				
**2**	–116.0	–103.9	–109.5	177.7	–45.5	–9.0	–25.8	68.2	–80.3	0.39	–0.39
		(35.4%)	(37.3%)		(15.5%)	(3.0%)	(8.8%)				
**3**	–109.6	–102.6	–120.3	196.9	–46.8	–10.4	–26.5	76.6	–83.7	0.41	–0.41
		(33.5%)^a^	(39.2%)		(15.3%)	(3.4%)	(8.6%)				
**4**	–101.7	–95.5	–106.3	175.0	–42.3	–7.5	–25.2	68.7	–75.0	0.45	–0.45
		(34.5%)	(38.4%)		(15.3%)	(2.7%)	(9.1%)				
**5**	–121.9	–121.5	–172.1	279.6	–67.7	–9.7	–30.6	107.5	–108.0	0.25	–0.25
		(30.2%)	(42.9%)		(16.9%)	(2.4%)	(7.6%)				
Three-body Fragmentation											
.Cl^–^											
**1**	–183.3	–152.8	–227.2	362.3	–60.9	–26.5	–78.3	135.1	–165.7		
		(27.9%)		(41.6%)	(11.2%)	(4.8%)	(14.4%)				
.TS^–^	–199.1	–169.0	–267.3	439.7	–76.1	–22.1	–104.3	172.4	–202.5		
**1**		(26.5%)	(41.8%)		(11.9%)	(3.5%)	(16.3%)				
											

a % of attractive int. (Δ*E*^elstat^ + Δ*E*^ex^ + Δ*E*^pol^ + Δ*E*^corr^ + Δ*E*^disp^).

b = Δ*E*^ex^ + Δ*E*^rep^.

c = Δ*E*^pol^ + Δ*E*^disp^ + Δ*E*^corr^.

### 3.1 Bonding Situations

The GKS-EDA results related to the interaction of the [2]catenane (**1**) and all applied spherical and nonspherical anions (Table 2) reveal that the Cl^–^ exhibits the most stable interaction among the spherical anions. The total interaction energy values are −115.1, −110.6, and −104.8 kcal mol^–1^ for **1**.Cl^–^, **1**.Br^–^, and **1**.I^–^, respectively. The primary contributors to these interactions originate from the electrostatic, Δ*E*^elstat^, and exchange energy, Δ*E*^ex^, components. In fact, the Δ*E*^elstat^ and Δ*E*^ex^ contributions are of notable significance and exhibit a comparable magnitude in the recognition of halide anions. The exchange contribution (ranges from −121.5 to −95.8 kcal.mol^–1^ on going from chloride to iodide) is slightly more stabilizing than the electrostatic one (ranges from −107.4 to −91.9 kcal.mol^–1^). The Hirshfeld population analysis corroborates the findings observed for the electrostatic and exchange terms, indicating that a portion of the anion electron density flows from the anions toward the [2]catenane structure, thereby reducing the initial +1(*e*) of the [2]catenane, which acquires net charge ranging from +0.38 to +0.47(*e*), while the anions display a reduction in negative charges (varying between −0.38 and −0.47(*e*)). Furthermore, for these spherical anions, the polarization component, Δ*E*^pol^, also plays a significant role ranging from −50.3 to −39.0 kcal mol^–1^, while the dispersion, Δ*E*^disp^, and correlation, Δ*E*^corr^, contributions are less significant, but not negligible. In relation to the halide anions considered (Cl^–^, Br^–^, and I^–^), the stabilizing contributions in GKS-EDA are observed to diminish as the system progresses from chloride to iodide, which can be attributed to the direct consequence of the interaction distances **d1**–**d4** increasing in accordance with the anionic radii.

In relation to the nonspherical anions (BF_4_^–^, HSO_4_^–^, MS^–^, and TS^–^), the GKS-EDA reveals that the tosylate anion exhibits the most stabilizing interaction energy with **1**, with a Δ*E*^tot^ = −123.4 kcal mol^–1^, where 40.5% of the total attractive interaction comes from the exchange energy term, while 32.3% stems from electrostatic and 17.2% stems from polarization. Dispersion and correlation contributions are less significant. On the other hand, it also reveals that BF_4_^–^ presents the weakest interaction with **1** a Δ*E*^tot^ value of −87.6 kcal mol^–^, in which 47.3% of the total attractive interaction comes from the electrostatic contribution, while 30.8% comes from the exchange and 11.8% comes from polarization. In the same way, dispersion and correlation present a less significant contribution to the total interaction energy. In the case of the most stabilizing interactions, which involve TS^–^ and MS^–^, the exchange contribution exceeds that of the electrostatic component. In contrast, the less stabilizing interaction involving BF_4_^–^ displays an opposite behavior, with the electrostatic component exhibiting a slight stabilization effect that surpasses that of the exchange component. This can be attributed to the weaker capacity of BF_4_^–^ to be stabilized by σ–hole and hydrogen bonds when compared with the other nonspherical anions under consideration.

The Hirshfeld population analysis indicates that the tosylate (TS^–^)and mesylate (MS^–^) anions, which demonstrate the most stabilizing interactions with **1**, donate a considerable amount of electronic charge to **1**, thereby reducing its +1(*e*) charge to 0.26 and 0.27(*e*), respectively. Indeed, the data indicate that both TS^–^ and MS^–^ exhibit a higher degree of distributed charge than Cl^–^. The aforementioned findings can be substantiated by data presented in Table 1, which illustrate that both TS^–^ and MS^–^ have not only favorable distances and angles with the cavity of **1** but also possess a larger number of hydrogen bond receptors in comparison to Cl^–^, which contributes to a higher charge distribution. With regard to the anion HSO_4_^–^ and BF_4_^–^ anions, it is noteworthy that both exhibited the lowest charge distribution values when interacting with **1**. In the case of BF_4_^–^, the amount of charge distributed is even less than that observed for the charge transfer of all spherical anions employed. In contrast, for HSO_4_^–^, the charge distribution is slightly higher than that observed in I^–^ (Table 2). In consideration of the data presented by GKS-EDA, it can be concluded that the anions Cl^–^ and TS^–^ exhibit the most stabilizing interactions with the [2]catenane binding pocket. Accordingly, these anions were selected for the evaluation of the impact of the nature of the σ–hole donors on the anion recognition. This was achieved by modifying the structure of **1**, resulting in the [2]catenanes **2**–**5** (Figure S1). As previously stated in the Introductionsection, the modification entails the substitution of the reference σ–hole donor iodine by bromine **2**, chlorine **3**, fluorine **4**, and tellurium (−Te–CH_3_) **5**.

The findings of GKS-EDA for both the reference [2]catenane **1** and the modified ones **2**–**5** are presented in the first block of Table 3, where a two-body fragmentation scheme is considered (fragment 1: [2]catenane and fragment 2: anion). The second block presents a three-body fragmentation scheme for **1**.Cl^–^ and **1**.TS^–^, in which fragment 1: hydrogen-bond donor moiety, is depicted in red in Figures 1 and S1; fragment 2: macrocycle containing the σ-hole donors and is shown in blue in Figures 1 and S1; fragment 3: represents the anions.

In analyzing the modifications to the structure of **1**, it can be observed that as one progresses from **1** to **4**, there is a gradual substitution of the iodine for a less polarizable halogen (Br, Cl, and F). This leads to a gradual reduction in the significance of the σ-hole as one progresses from I to F. The EDA results confirm that changing from a stronger σ–hole donor (I) to a weaker σ–hole donor (F) will have a considerable impact on anion interaction, thereby demonstrating that the halogen bonds formed between the [2]catenane and the anion play a pivotal role.

To illustrate, the gradual transition from **1**.Cl^–^ to **4**.Cl^–^ not only entails a reduction in electrostatic components from −107.4 to −92.6 kcal mol^–1^, but also in other terms, namely exchange (from −121.5 to −78.4 kcal mol^–1^), and polarization (−50.3 to −27.6 kcal mol^–1^), while the correlation and dispersion terms are less affected. As a result of these destabilizing effects, the total interaction energy was significantly reduced, confirming that the halogen-triazole and consequently the halogen bonds they form with the anions under consideration are crucial to the anion recognition process. Comparative analysis of the trends observed in the values of Δ*E*^pauli^ and Δ*E*^oi^ reveals a clear correlation between the transition from **1** to **4** and the loosening of orbital stabilization, which in turn results in a decline in Pauli repulsion. A notable reduction in the interaction energy between the [2]catenane and the anions in question, chloride or tosylate, is observed when the halogen bond donor is changed from iodine to fluorine. This reduction is observed regardless of the nature of the anion, whether spherical or nonspherical. The weakening of the halogen bond donor impairs the interaction energies and, consequently, the anion recognition. Conversely, when the halogen bond donor is replaced with a bulky chalcogen group, the stabilization initially observed with iodine is restored. Therefore, our findings support the conclusion that both strong halogen and chalcogen bond donors are essential for the recognition of anions regardless of their shape. The analysis of the Hirshfeld populations serves to corroborate the trends observed in the stability of the interactions of the reference and modified [2]catenanes (**1**–**5**), in which iodine, bromine, chlorine, fluorine, and tellurium-methyl groups are used as halogen or chalcogen bond donor centers. The results demonstrate that the most effective donors also exhibit the highest charge influxes from the anions, resulting in a significant charge reorganization. This, in turn, leads to enhanced stabilization in the exchange, polarization, and electrostatic components.

### 3.2 **σ**-Hole vs H-Bond vs Mechanical Bond Contributions

**Figure 4:**
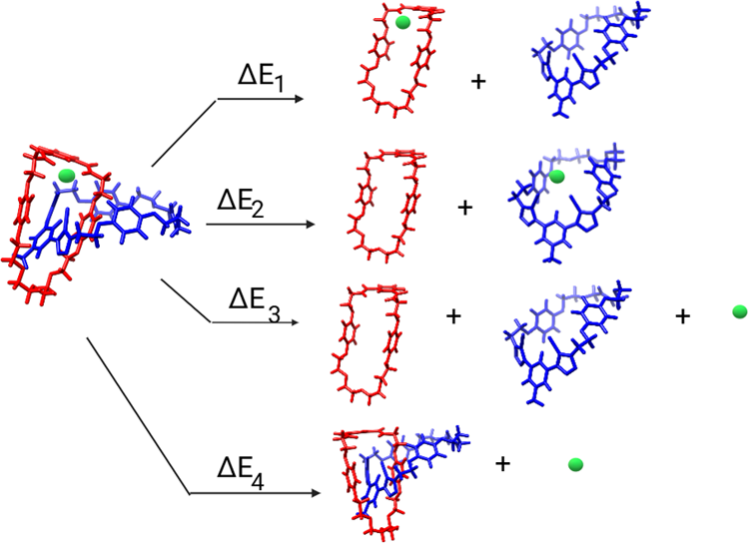
Homodesmostic reaction scheme to obtain the (**1**) σ–hole contribution to the interaction with the applied anion, the (**2**) H-bond contribution to the interaction with the applied anion, the energy required to separate all three components of the studied system (**3**), and the (**4**) energy to remove the anion from the MIM.

**Figure 5:**
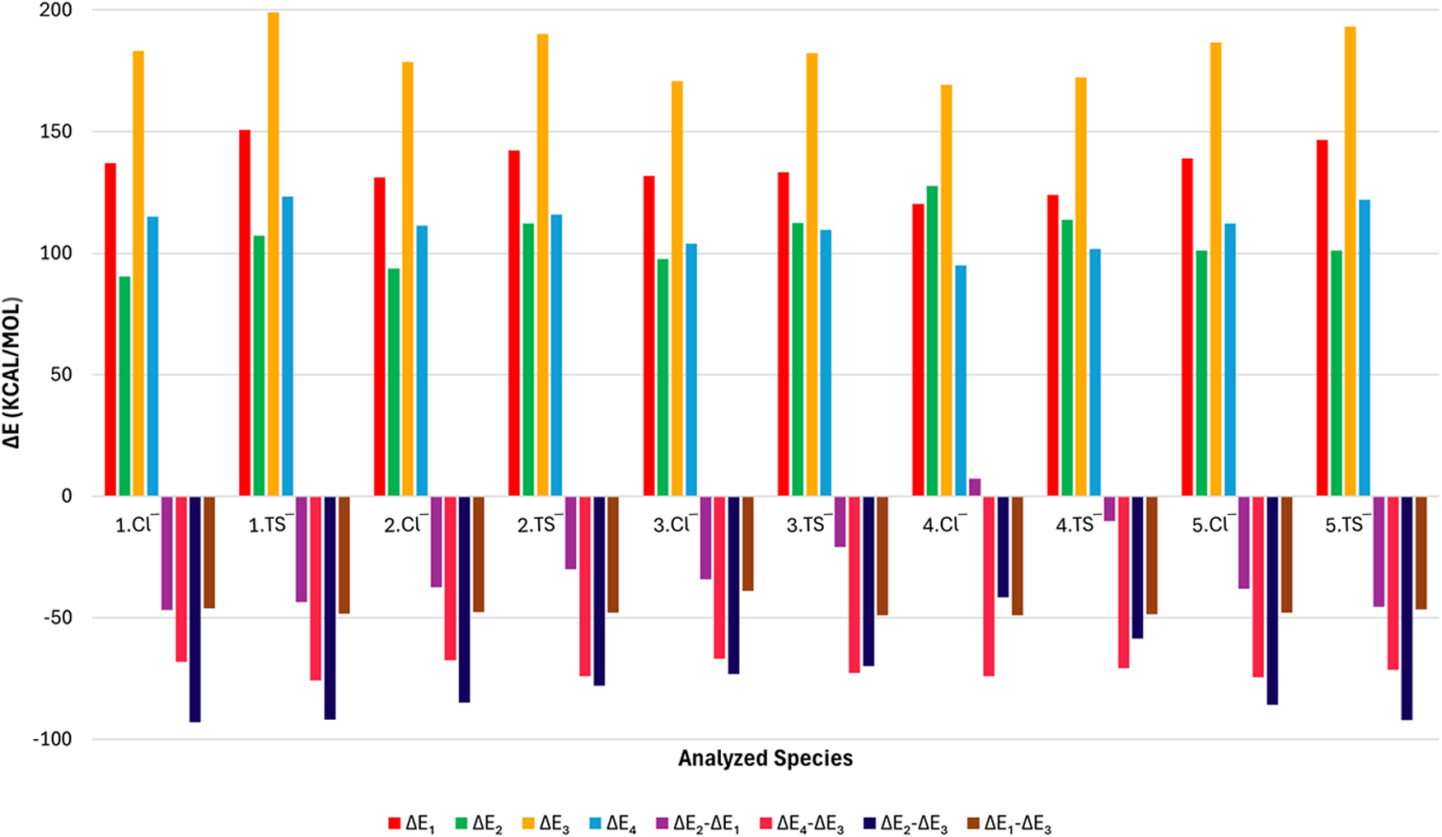
Contributions (kcal.mol^–1^) of the σ–hole interaction and H-bonds interaction on the applied anions (Cl^–^ and TS^–^) as they interact with the [2]catenanes **1**–**5** following the scheme presented in Figure 4.

To gain deeper insight into the contributions of hydrogen bonds, σ–hole interactions, and mechanical bonds to anion recognition by each considered structure (**1**–**5**), a different fragmentation scheme (Figure 4) has been employed without any further change to the optimized geometries (Table 1). The presented results pertain to the [2]catenane structures in interaction with either Cl^–^ or TS^–^ anions. The results obtained from each contribution are listed in Figure 5. According to the fragmentation scheme adopted in Figure 4, while Δ*E*_1_ provides insight into the contribution of the portion containing the σ–hole, Δ*E*_2_, in contrast, elucidates the contribution of the portion containing the hydrogen bond donors to the stabilization of the anion when it interacts with the [2]catenane. Finally, Δ*E*_3_ and Δ*E*_4_ values represent the energy required to separate the three interacting fragments (red: H-bond donors; blue: σ–hole donors; green: anion, whether spherical or not) and the energy to separate the applied anion from interacting with the selected [2]catenane structure, respectively. These two components can be employed to demonstrate the influence of the mechanical bond on anion recognition.

It is evident that when the chloride anion is taken into consideration, the primary contributor to the overall stabilization is the σ–hole interaction, as evidenced by the considerably higher value of Δ*E*_1_ in comparison to Δ*E*_2_. This behavior is observed in all structures except for **4**, which contains fluorine atoms and thus exhibits the least favorable σ–hole donor properties. Consequently, in **4**.Cl^–^, the main contribution comes from the H-bond interaction with the anion. Despite the fact that the chloride anion would remain situated at a greater depth within the [2]catenane’s cavity due to its relatively small atomic radius, the magnitude of the σ–hole interaction appears to be the crucial factor. With regard to Δ*E*_3_ values, it is evident that for both anions, Cl^–^ and TS^–^, it is still more endothermic to separate all of the structure’s components into three fragments. However, the energy penalty, Δ*E*_4_, to separate the anion from the [2]catenane cavity must be smaller than Δ*E*_3_, since the mechanical bond is maintained.

With regard to the tosylate anion, as with chloride, the σ–hole interaction is the primary contributor to overall stabilization. Indeed, the higher σ–hole contribution observed for all [2]catenanes, including **4**, is not exclusive to the interaction with the tosylate. Additionally, the contribution from hydrogen bonds is markedly higher than that observed for chloride, confirming that not only is there a significant orbital overlap between the tosylate anion and the halogen bond donors, but also that the tosylate anion is capable of forming hydrogen bonds with greater efficacy than chloride. The difference between the tosylate and chloride anions is that the tosylate is closer to the halogen bond donors, allowing significant orbital overlap, as illustrated in Figure S2. Moreover, the tosylate anion contains a greater number of hydrogen bond receptors than chloride, thereby enabling a substantial orbital overlap with hydrogen bond donors.

Alchemical free energy principles have been employed to calculate the free energy differences associated with the anion transfer processes, as represented in Figure 4, by subtracting the Δ*E_n_* values, thereby allowing for a more nuanced understanding of the interplay between the various contributions involved in anion recognition. The subtraction of the Δ*E_n_* values is plotted in Figure 5. For instance, Δ*E*_2_ – Δ*E*_1_ values demonstrate the enhanced stability achieved when the anion is transferred from an environment that stabilizes it predominantly through hydrogen bonds to one that provides stabilization exclusively through halogen/chalcogen bonds, specifically the σ–hole donor portion of the molecule. Such stabilization is slightly more significant to Cl^–^ than to TS^–^, since the change of environment is in agreement with our previous findings. The stabilization effect of TS^–^ is less pronounced than that of Cl^–^, which can be attributed to the greater role played by hydrogen bonds in the overall stabilization of tosylate given the abundance of hydrogen bond receptors present in it. The most unusual situation is observed by comparing **4**.Cl^–^ and **4**.TS^–^, where Δ*E*_2_ – Δ*E*_1_ results in a faint gain in stabilization for **4**.TS^–^ and a decrease in stabilization for **4**.Cl^–^ (positive value). This particular finding is expected since the main contribution for **4**.Cl^–^ comes mainly from the H-bonds donor portion, while for TS^–^, as demonstrated in Figure S2, the shorter distances between the fluorine atoms and TS^–^, allow it to obtain a more stabilizing σ–hole interaction.

Δ*E*_2_ – Δ*E*_3_ illustrates the stability gained when all of the interacting species are separated from each other and when the anion is introduced to interact solely with the σ–hole donor portion. It can be directly contrasted with Δ*E*_1_ – Δ*E*_3_, which shows the enhanced stability that is gained when all components are separated and when the anion interacts exclusively with the H-bond donor portion. Notice that Δ*E*_2_ – Δ*E*_1_ = (Δ*E*_2_ – Δ*E*_3_) – (Δ*E*_1_ – Δ*E*_3_). As has been demonstrated, the results confirm that the primary gain in stability is achieved when the anions are in contact with the σ–hole interaction environment.

Ultimately, the role of the mechanical bond in the anion interaction is determined by Δ*E*_4_ – Δ*E*_3_ values, which quantify the stability gained when the separated components of [2]catenane (red and blue) are disentangled while maintaining their original geometry. In all cases, the contribution of the mechanical bond is stabilizing, providing ca. −70 to −80 kcal.mol^–1^ of stabilization. For instance, concerning the chloride anion, the contribution of the mechanical bond exhibits a negligible decrease in stability as the σ–hole donor strength declines from **1** to **3.** The exception is **4**.Cl^–^. This structure exhibits a higher gain of stability through the mechanical bond, which compensates for the lack of stability of the σ–hole interaction provided by fluorine. A similar gain of stability was observed in **5**.Cl^–^. This structure contains groups Te–CH_3_ as σ–hole donors, which due to their size confer to the moieties a different conformational degree, which is likely responsible for the observed differences.

With regard to the interactions with the anion TS^–^, a comparable trend is evident with regard to the contribution of the mechanical bonds, albeit with a slight stabilizing effect in comparison with those observed for Cl^–^. The outlier in these trends is **5**, wherein the stabilizing effect of the mechanical bond is observed to be diminished when tosylate is interacting as opposed to when chloride is engaged in the process. In order to comprehend the underlying mechanism, it is essential to undertake a comparative analysis of both **5**Cl^–^ and **5**TS^–^ with respect to the impact of the applied anions on the [2]catenane’s structure. The largest root-mean-square deviation, RMSD = 1.80, is obtained when contrasting the optimized geometries of **5**.Cl^–^**5**.TS^–^. The data indicate a notable variation in the [2]catenane framework when different anions are employed. Given that tellurium is a bulky atom and that methyl groups are in closer proximity to TS^–^ than to Cl^–^, as evidenced by the distances depicted in Figure S3 the observed variation is to be expected. It can be surmised that the incorporation of Cl^–^ into structure **5** results in a structure with a higher degree of organization, allowing for better accommodation of the anion and leading to a greater stability gain from the mechanical bond. In comparison, the resulting structure when applying TS^–^ causes significant conformational changes to the [2]catenane’s structure due to the repulsion, due to the short distances between TS^–^ and Te–CH_3_ group. This results in distortion of the [2]catenane and a reduction in the stability gained from the mechanical bond.

## 4 Usage of Counterion

The presence of a counterion, PF_6_^–^ (Figure S4), and its effects on the physical terms GKS-EDA have been taken into account as presented in Table 4, in which the GKS-EDA outcomes for **1**.Cl^–^ and **6**.Cl^–^ are reported. It is noticeable a significant decrease in the value of Δ*E*^tot^ with the addition of the counterion to the [2]catenane interacting with the anion (**1**.Cl^–^: Δ*E*^tot^ = −115.1 kcal mol^–^; **6**.Cl^–^: Δ*E*^tot^ = −86.5 kcal mol^–^). While previously for **1**.Cl^–^ there’s a positively charged [2]catenane interacting with a negatively charged anion, resulting in a neutral system. The introduction of the counterion imbues the entire system with a net negative charge, thereby reducing the electrostatic potential energy value. In contrast to the interaction between the anion and **1**, which results in direct charge neutralization, the anion’s interaction with **6** does not lead to the same outcome. As previously reported, the most stabilizing contributions in **1**.Cl^–^ stem from Δ*E*^ex^, with 40.2% of the total attractive interaction, followed by Δ*E*^elst^ with 35.6%. However, in the case of **6**.Cl^–^, Δ*E*^ex^ does not exhibit a significant decrease in its contribution, but, in fact, the presence of the counterion increases the percent contribution 44.4%. On the other hand, Δ*E*^elstat^ exhibits a 6.8% deficit in the contribution for the overall attractive interaction. The depletion in the Δ*E*^elstat^ contribution is compensated by the percent increase in Δ*E*^corr^, Δ*E*^pol^, and Δ*E*^ex^.

**Table 4:** Results Obtained from the GKS-EDA Where the Two Analyzed Fragments Were the Overall Neutral Structure ([2]Catenane + Counteranion) as Fragment 1, and the Applied Spherical Anion as Fragment 2

	Δ*E*^tot^	Δ*E*^elstat^	Δ*E*^ex^	Δ*E*^rep^	Δ*E*^pol^	Δ*E*^corr^	Δ*E*^disp^	Δ*E*^pauli^^b^	Δ*E*^oi^^c^
**1**.Cl^–^	–115.1	–107.4	–121.5	186.9	–50.3	–15.8	–7.0	65.4	–73.0
		(35.6%)^a^	(40.2%)		(16.7%)	(5.2%)	(2.3%)		
**6**.Cl^–^	–86.5	–78.9	–121.7	187.3	–51.0	–15.2	–7.0	65.6	– 73.2
		(28.8%)	(44.4%)		(18.6%)	(5.6%)	(2.6%)		

a % of attractive int. (Δ*E*^elstat^ + Δ*E*^ex^ + Δ*E*^pol^ + Δ*E*^corr^ + Δ*E*^disp^).

b = Δ*E*^ex^ + Δ*E*^rep^.

c = Δ*E*^pol^ + Δ*E*^disp^ + Δ*E*^corr^.

## 5 Conclusions

The present study elucidated the role of both hydrogen and halogen bonds, from an electronic structure perspective, in the anion recognition process by the [2]catenane, **1**, which contains a macrocycle with two hydrogen bond donors entangled with another macrocyclic component containing the bis-iodo-triazole pyridinium moiety as a halogen bond donor. Spherical (Cl^–^, Br^–^, and I^–^) and nonspherical anions (BF_4_^–^, HSO_4_^–^, mesylate MS^–^, and tosylate TS^–^) have been considered. The role of different σ–hole donors has also been considered. The structure of **1** was modified by incorporating other σ–hole donors, namely bromine, chlorine, fluorine, as well as −Te–CH_3_ as a chalcogen bond donor, leading to the modified [2]catenanes **2**–**5**. Insights into anion recognition were gained by quantifying the contributions of not only the mechanical but also hydrogen and halogen/chalcogen bonds to anion recognition using the GKS-EDA energy partition scheme.

The minimum structures and their geometric parameters demonstrate that both classes of anions are stabilized by both hydrogen bonds and halogen/chalcogen bonds. Additionally, these parameters are dependent on the minimum conformation adopted by the [2]catenane and the nature of the interacting anion. For example, the spherical anion, Cl^–^, was identified as the most effective interacting anion, exhibiting a deeper penetration into the [2]catenane’s active site. In the case of BF_4_^–^, HSO_4_^–^, mesylate MS^–^, and tosylate TS^–^ anions, they are mainly stabilized by hydrogen bonds when compared with the spherical ones. TS^–^ and MS^–^ present the most stabilizing interactions with **1**, as revealed by the geometric parameters, red shifts in the stretching frequencies of NH_1_ and NH_2_ groups, also confirmed by the GKS-EDA findings.

The GKS-EDA results showed that the stabilizing contributions are observed to diminish as the system progresses from chloride to iodide, which can be attributed to the direct consequence of the interaction distances **d1**–**d4** increasing in accordance with the anionic radii. Concerning the nonspherical anions, the GKS-EDA confirms that the tosylate anion exhibits the most stabilizing interaction energy, followed by the mesylate, while BF_4_^–^ exhibits the least stabilizing interaction. In consideration of the data presented by the GKS-EDA, it can be concluded that the anions Cl^–^ and TS^–^ exhibit the most stabilizing interactions with the **1** binding pocket. Accordingly, these anions were selected for the evaluation of the impact of the nature of the σ-hole donors on anion recognition. This was achieved by modifying the structure of **1**, resulting in [2]catenanes **2**–**5**. The GKS-EDA results confirm that changing from a stronger σ-hole donor (I) to a weaker σ-hole donor (F) will have a considerable impact on the anion interaction, thereby demonstrating that the halogen bonds formed between the [2]catenane and the anion play a pivotal role. The weakening of the halogen bond donor impairs the interaction energies and consequently the anion recognition. Conversely, when the halogen bond donor is replaced with a bulky chalcogen group, the stabilization initially observed with iodine is restored. The introduction of the counterion PF_6_^–^ imbues the entire system with a net negative charge, thereby reducing the electrostatic potential energy value. In contrast to the interaction between the anion and **1**, which results in direct charge neutralization, the anion’s interaction with **6** does not lead to the same outcome but shows a significant decrease in the value of Δ*E*^tot^.

## Supporting Information Available

3D visual representation of the modified [2]catenanes (**2**–**5**) (Figure S1); comparison distances between **4**.Cl^–^ and **4**.TS^–^ (Figure S2); comparison distances between **5**.Cl^–^ and **5**.TS^–^ (Figure S3); 3D visual representation of the modified counterion containing [2]catenane (**6**) (Figure S4); values referencing the hydrogen bond, σ-hole, and mechanical bond contributions in Figure 5 (Table S1); Cartesian coordinates of **1**–**6**.Cl^–^, **1**.Br^–^, **1**.I^–^, **1**.BF_4_^–^, **1**.HSO_4_^–^, **1**.MS^–^, and **1**–**5**.TS^–^ (Tables S2–S17) (PDF).
