# Supplementary material for: Exploring the Capability of Mechanically Interlocked Molecules in Anion Recognition: A Computational Insight
Source: ACS Phys Chem Au. 2024 Dec 10;5(1):101–11. doi: 10.1021/acsphyschemau.4c00089 (PMC11758374; doi:10.1021/acsphyschemau.4c00089)
Supplement: Supplementary file 1 — pg4c00089_si_001.pdf [file pg4c00089_si_001.pdf]

Supporting Information: Exploring the Capability of Mechanically Interlocked Molecules in Anion Recognition: A Computational Insight. †

Fábio J. Amorim and Giovanni F. Caramori*

Departamento de Química, Universidade Federal de Santa Catarina, Campus Universitário Trindade, 88040-900, Florianópolis, SC, Brazil.

E-mail: giovanni.caramori@ufsc.br

Contents

Illustrations **S-3**

Hydrogen Bond vs σ-hole vs Mechanical bond contributions **S-7**

Cartesian Coordinates **S-8**

Supporting Information Available

In a nutshell, the first section of the SI available includes the illustrations referencing all modified structures **2-5** in S1, the distances of the applied Cl^- and Ts^- with the $\sigma-$hole donors in **4**, S2, the distances of the applied Cl^- and Ts^- with the $\sigma-$hole donors in **5**, S3, and the [2]catenane structure alongside a counter ion in **6**, S4. The second section references to the table S1 with the obtained contribution values utilized for the Hydrogen bond, σ-hole and Mechanical bond presented as a graph labelled as **FIGURE 5** in the paper and the second section provides the Cartesian Coordenates of the obtained optimized structures, utilized for the analysis, of all studied system.

Illustrations

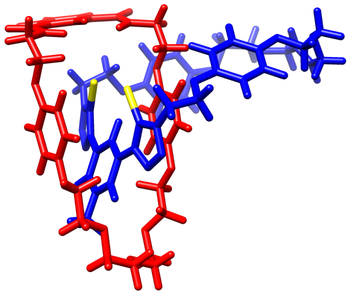

2

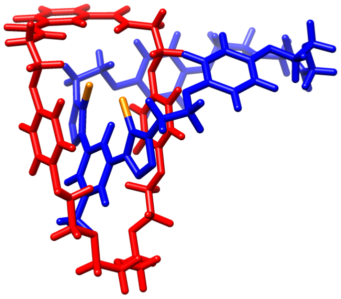

3

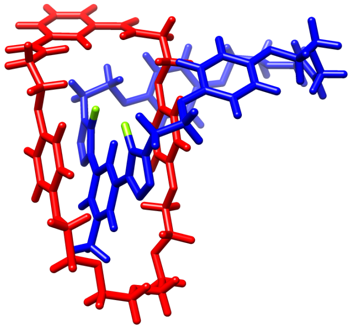

4

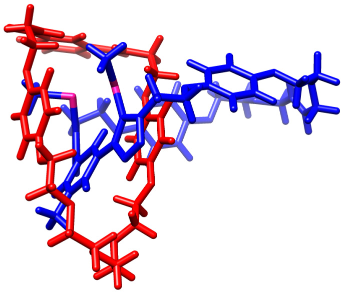

5

Figure S1: Modified [2]Catenanes **2**-**5** where the halogen bond donor iodine atoms in (**1**) have been replaced by: (**2**) yellow - bromine; (**3**) orange - chlorine; (**4**) green - fluorine; (**5**) pink - tellurium ($-$Te$-CH_3$).

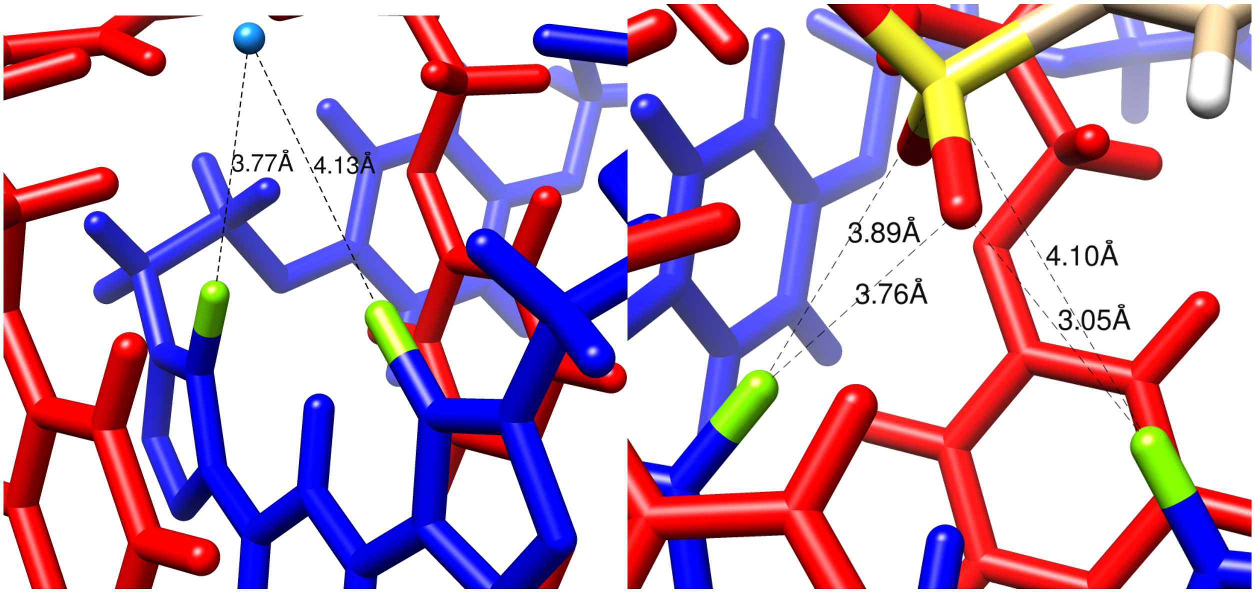

(a) **4**.Cl^{-} (b) **4**.TS^{-}

Figure S2: **a**) Distance of Cl^{-} in relation to the halogen bond donors Flourines in **4**.Cl^{-}; **b**) Distances of TS^{-} (including Oxygen atoms and Sulfur center atom) in relation to the halogen bond donor Flourines in **4**.TS^{-}

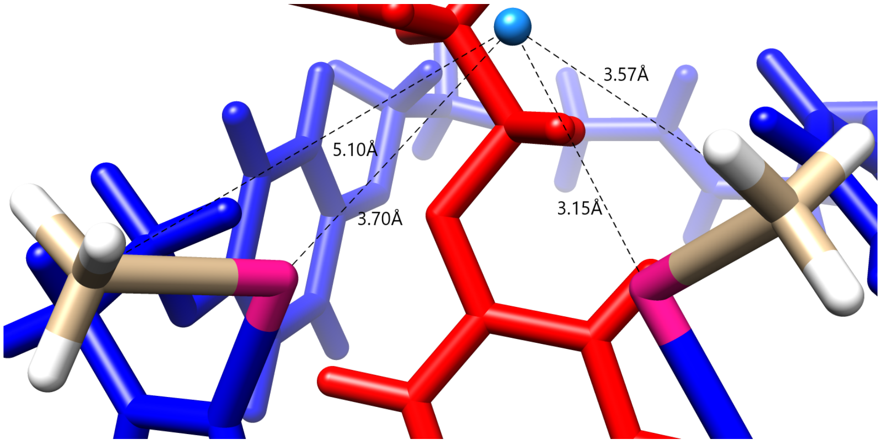

(a) **5**.Cl$^-$

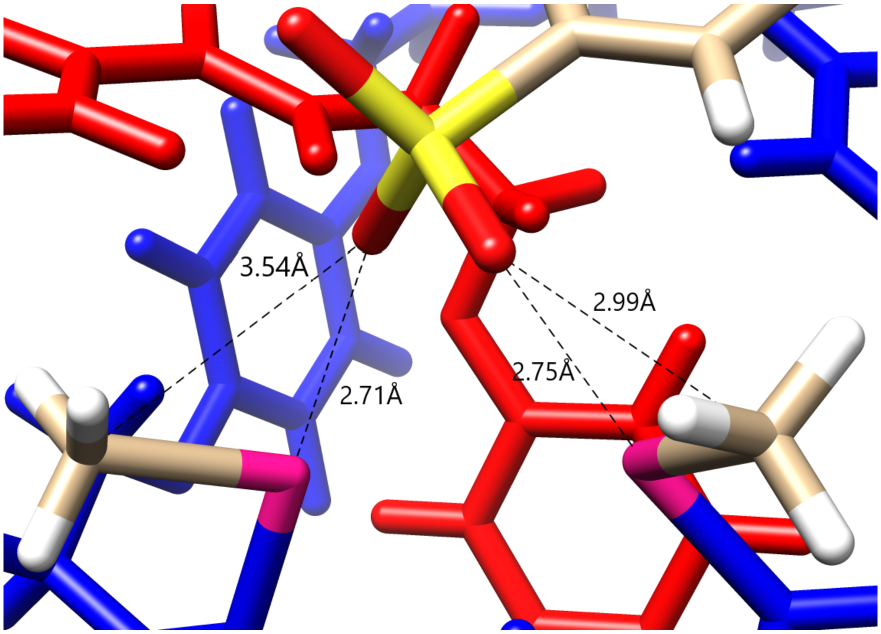

(b) **5**.TS$^-$

Figure S3: Distance of **a**) Cl$^-$ in **5**.Cl$^-$ and **b**) TS$^-$ in **5**. TS$^-$ in relation to the halogen bond donors atoms of Tellurium and the Methyl groups

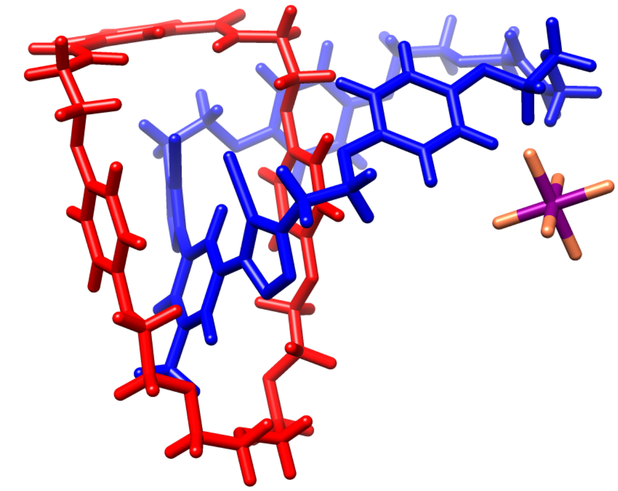

6

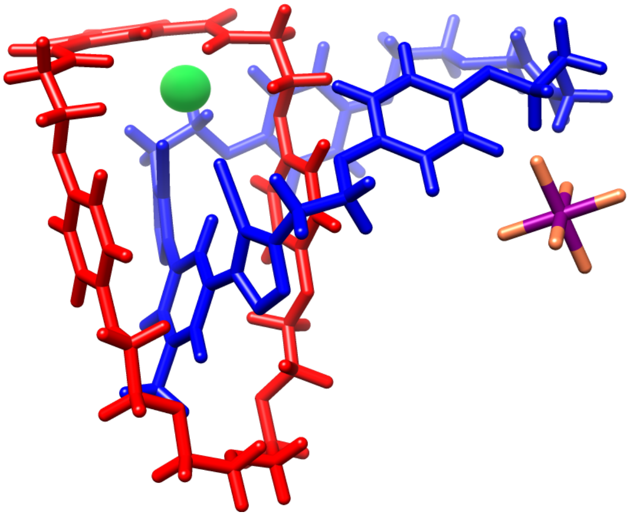

6.X$^-$

Figure S4: [2]Catenane in the presence of the counter ion $PF_6{}^-$, **6**

Table of Hydrogen bond vs σ-hole vs Mechanical bond contributions

Table S1: Values containing the contributions of the Hydrogen bond, $\sigma-$hole interaction, and Mechanical bond, in accordance to the proposed fragmentation schemes (from ΔE_1 to ΔE_4)

	ΔE_1	ΔE_2	ΔE_3	ΔE_4	$(\Delta E_2 - \Delta E_1)$	$(\Delta E_4 - \Delta E_3)$	$(\Delta E_2 - \Delta E_3)$	$(\Delta E_1 - \Delta E_3)$
**1**.Cl^-	137.1	90.4	183.3	115.1	-46.7	-68.2	-92.9	-46.2
**1**.TS^-	150.8	107.3	199.1	123.4	-43.5	-75.7	-91.8	-48.3
**2**.Cl^-	131.1	93.7	178.7	111.3	-37.4	-67.4	-85.0	-47.6
**2**.TS^-	142.3	112.2	190.1	116.0	-30.1	-74.1	-77.9	-47.8
**3**.Cl^-	131.8	97.7	170.8	104.0	-34.1	-66.8	-73.1	-39.0
**3**.TS^-	133.3	112.4	182.3	109.6	-20.9	-72.7	-69.9	-49.0
**4**.Cl^-	120.3	127.6	169.2	95.1	7.3	-74.1	-41.6	-48.9
**4**.TS^-	123.9	113.8	172.4	101.7	-10.1	-70.7	-58.6	-48.5
**5**.Cl^-	139.0	101.0	186.8	112.3	-38.0	-74.5	-85.8	-47.8
**5**.TS^-	146.7	101.2	193.3	121.9	-45.5	-71.4	-92.1	-46.6

Cartesian Coordinates

Table S2: Cartesian Coordenates of **1**.Cl^{-}

**1**.Cl^{-}	x	y	z
N	0.550436	-0.042347	-6.048427
C	1.631566	-0.317126	-5.259650
C	1.454455	-0.599174	-3.886506
C	0.145660	-0.590445	-3.351461
C	-0.962010	-0.303282	-4.188299
C	-0.725250	-0.034187	-5.549480
C	0.782035	0.196845	-7.502177
C	2.655624	-0.832177	-3.082681
N	3.880975	-0.307874	-3.512893
N	4.846444	-0.638958	-2.630467
N	4.218098	-1.394699	-1.617046
C	2.865648	-1.527097	-1.865920
I	1.531406	-2.625563	-0.660141
C	5.049492	-1.878683	-0.501863
C	5.158096	-0.867363	0.640024
O	3.889641	-0.920826	1.389328
C	3.809900	-0.164212	2.576252
C	4.769012	0.792483	2.980494
C	4.593680	1.497351	4.197436
C	3.460857	1.241743	5.007165
C	2.497247	0.283039	4.592579
C	2.671732	-0.417534	3.386164
O	3.203853	1.870980	6.239226

C	4.163447	2.907830	6.676036
C	3.695501	3.431224	8.031570
O	2.426254	4.171565	7.983153
C	2.560836	5.536165	7.453925
C	1.191425	6.143517	7.279855
C	0.025797	5.493931	7.530211
C	-1.339287	6.095568	7.333475
O	-2.052907	5.228072	6.363044
C	-3.508338	5.451977	6.359767
C	-4.119494	4.436924	5.410862
O	-3.899616	4.911796	4.026403
C	-4.194561	4.009772	2.985710
C	-4.011637	4.509048	1.668354
C	-4.253540	3.678375	0.558855
C	-4.673969	2.338243	0.755929
C	-4.864539	1.837449	2.061932
C	-4.630517	2.676523	3.177779
O	-4.873255	1.580952	-0.422512
C	-5.101996	0.135685	-0.243018
C	-5.356493	-0.461189	-1.628387
N	-4.243808	-0.246421	-2.569857
N	-4.397384	0.693832	-3.610389
N	-3.223830	0.711641	-4.282624
C	-2.332708	-0.189153	-3.685418
C	-2.984418	-0.806193	-2.593901
I	-2.320822	-2.272171	-1.242493
H	2.607220	-0.328145	-5.754402

H	-0.010277	-0.762990	-2.286830
H	-1.530719	0.194805	-6.247136
H	0.902616	-0.778380	-8.000704
H	-0.084459	0.729216	-7.919795
H	1.695136	0.801996	-7.619873
H	6.054539	-2.062790	-0.913875
H	4.634318	-2.830547	-0.133915
H	5.337377	0.151335	0.247249
H	5.996673	-1.155864	1.302613
H	5.652364	1.003549	2.371377
H	5.348544	2.230269	4.494380
H	1.626651	0.092212	5.227781
H	1.943488	-1.169570	3.066775
H	5.171917	2.461234	6.789511
H	4.213932	3.712342	5.917061
H	4.500142	4.065587	8.460166
H	3.508736	2.584487	8.713877
H	3.178522	6.150187	8.147729
H	3.084379	5.531461	6.472784
H	1.184219	7.178725	6.907916
H	0.051364	4.459264	7.892556
H	-1.907982	6.105010	8.290019
H	-1.282347	7.135011	6.948513
H	-3.751945	6.487700	6.043435
H	-3.917977	5.289286	7.379992
H	-5.207492	4.333789	5.590429
H	-3.630233	3.456401	5.564804

H	-3.674319	5.541083	1.528992
H	-4.108181	4.052182	-0.459713
H	-5.160199	0.801404	2.251411
H	-4.788615	2.267544	4.179050
H	-6.002302	-0.042340	0.372744
H	-4.222793	-0.314405	0.253187
H	-6.242895	0.001537	-2.089494
H	-5.533155	-1.544863	-1.520692
C	-7.069582	-3.573993	0.502964
C	-6.394592	-4.677580	-0.063594
C	-5.044272	-4.941659	0.280865
C	-4.384463	-4.092393	1.200881
C	-5.043562	-2.963850	1.742266
C	-6.397270	-2.715776	1.399759
C	-4.362191	-6.113309	-0.384078
O	-5.041795	-6.990225	-1.030809
N	-2.993591	-6.171870	-0.282450
C	-2.201474	-7.211662	-0.956116
C	-1.001141	-6.613005	-1.688483
O	-1.532941	-5.739269	-2.756159
C	-0.597161	-5.058137	-3.552279
C	0.805414	-5.163052	-3.404361
C	1.665340	-4.457656	-4.280901
C	1.119902	-3.659718	-5.312132
C	-0.287190	-3.539122	-5.447303
C	-1.140474	-4.228183	-4.569047
O	1.889310	-2.959709	-6.265553

C	3.349914	-3.189016	-6.232582
C	3.963791	-2.488082	-7.428132
O	4.001753	-1.038977	-7.170602
C	4.605537	-0.308941	-8.299488
C	4.838354	1.132447	-7.888047
O	3.541351	1.824057	-7.786487
C	3.714328	3.216708	-7.343400
C	2.342747	3.862525	-7.163483
O	1.494559	3.228764	-6.139381
C	2.131463	3.166309	-4.812426
C	0.988301	2.973865	-3.819891
O	1.610433	2.487954	-2.573827
C	0.741990	2.004461	-1.575644
C	-0.672927	2.060350	-1.658398
C	-1.455856	1.506966	-0.625642
C	-0.835963	0.906057	0.494780
C	0.579675	0.879903	0.592513
C	1.363205	1.426762	-0.444741
O	-1.700746	0.363793	1.464715
C	-1.068644	-0.404438	2.556901
C	-2.185521	-1.009781	3.406375
N	-2.983227	-1.983068	2.645791
C	-4.355234	-1.974826	2.648325
O	-5.039583	-1.139896	3.347857
H	-8.113248	-3.376451	0.237551
H	-6.895262	-5.345708	-0.771388
H	-3.358056	-4.316181	1.507051

H	-6.902397	-1.851772	1.843119
H	-1.818321	-7.946926	-0.220802
H	-2.864922	-7.745183	-1.656675
H	-0.371680	-6.017439	-0.999159
H	-0.392334	-7.424700	-2.132165
H	1.247476	-5.780980	-2.619013
H	2.745928	-4.547169	-4.142746
H	-0.699384	-2.919429	-6.249198
H	-2.227251	-4.145533	-4.667000
H	3.777393	-2.799770	-5.288887
H	3.551696	-4.275613	-6.301406
H	4.999131	-2.863673	-7.571957
H	3.379906	-2.704734	-8.346458
H	5.583165	-0.768346	-8.558067
H	3.943902	-0.365502	-9.188705
H	5.468375	1.631976	-8.654508
H	5.367579	1.173647	-6.913490
H	4.278854	3.792853	-8.108450
H	4.297846	3.239557	-6.399858
H	2.497967	4.933923	-6.912245
H	1.755999	3.794265	-8.094995
H	2.829780	2.308595	-4.753252
H	2.692031	4.099957	-4.603547
H	0.446270	3.919785	-3.631110
H	0.274535	2.227668	-4.211466
H	-1.175298	2.514173	-2.516259
H	-2.546765	1.529751	-0.693465

H	1.081267	0.446842	1.460084
H	2.455745	1.398167	-0.387838
H	-0.452575	0.266604	3.185366
H	-0.421890	-1.194475	2.126219
H	-2.865848	-0.226586	3.777446
H	-1.714543	-1.496478	4.283434
H	-2.451553	-5.427162	0.186556
H	-2.450604	-2.645472	2.057502
Cl	-0.824355	-3.993454	0.997429

Table S3: Cartesian Coordenates of **2**.Cl^-

**1**.Br^-	x	y	z
N	0.583782	-0.036628	-5.830932
C	1.660957	-0.333814	-5.042559
C	1.467858	-0.714845	-3.696242
C	0.153353	-0.753020	-3.179030
C	-0.947376	-0.444783	-4.013132
C	-0.699285	-0.095887	-5.355015
C	0.834399	0.290458	-7.265522
C	2.643389	-1.028396	-2.886517
N	3.910892	-0.593335	-3.286918
N	4.837155	-1.022660	-2.402698
N	4.140340	-1.751907	-1.415461
C	2.788678	-1.764410	-1.687395
C	4.911218	-2.323013	-0.297558
C	5.112267	-1.321387	0.839657
O	3.829693	-1.231098	1.559143

C	3.786717	-0.393493	2.693145
C	4.813842	0.503891	3.064713
C	4.662179	1.310252	4.220178
C	3.486572	1.212997	5.002836
C	2.459577	0.305642	4.626525
C	2.609713	-0.493690	3.480021
O	3.244186	1.956821	6.172235
C	4.246673	2.980934	6.536793
C	3.765837	3.666090	7.813842
O	2.519120	4.428724	7.659553
C	2.689328	5.708756	6.957878
C	1.338611	6.347360	6.750910
C	0.159187	5.784071	7.118499
C	-1.189344	6.411311	6.889359
O	-1.963136	5.465963	6.045305
C	-3.416018	5.702708	6.102666
C	-4.081234	4.622076	5.268571
O	-3.953891	5.002330	3.843880
C	-4.241110	4.015390	2.881892
C	-4.120847	4.428082	1.527861
C	-4.352353	3.510296	0.487443
C	-4.700009	2.169392	0.790970
C	-4.834927	1.754573	2.134001
C	-4.612119	2.681968	3.180623
O	-4.887957	1.321721	-0.327143
C	-5.016922	-0.118760	-0.041685
C	-5.273981	-0.824366	-1.375248

N	-4.209323	-0.582472	-2.363895
N	-4.454027	0.290137	-3.446797
N	-3.293511	0.376138	-4.136667
C	-2.320212	-0.413822	-3.513885
C	-2.907665	-1.025620	-2.386702
H	2.643433	-0.283163	-5.520323
H	-0.013543	-0.993596	-2.129165
H	-1.501375	0.147594	-6.051502
H	1.084491	-0.645480	-7.790106
H	-0.072808	0.739509	-7.693723
H	1.679448	0.994968	-7.331898
H	5.892835	-2.614220	-0.703648
H	4.396487	-3.224771	0.070262
H	5.409842	-0.334098	0.438693
H	5.901827	-1.692461	1.521219
H	5.731538	0.593436	2.477052
H	5.468214	1.997055	4.491803
H	1.557206	0.234690	5.241761
H	1.832612	-1.205181	3.184496
H	5.224428	2.497355	6.736754
H	4.368860	3.695596	5.699874
H	4.581091	4.322350	8.186532
H	3.541372	2.906559	8.582176
H	3.355026	6.377855	7.549005
H	3.176091	5.557837	5.969383
H	1.357053	7.327291	6.251386
H	0.159145	4.802649	7.607659

H	-1.726243	6.552975	7.853677
H	-1.108434	7.396008	6.383880
H	-3.672190	6.712227	5.718738
H	-3.769345	5.624501	7.153333
H	-5.154727	4.528126	5.523842
H	-3.575440	3.656572	5.458506
H	-3.837283	5.462069	1.307032
H	-4.253397	3.816467	-0.558874
H	-5.086599	0.723657	2.402314
H	-4.727732	2.339796	4.212505
H	-5.886220	-0.307002	0.613022
H	-4.095390	-0.473181	0.456487
H	-6.205087	-0.454326	-1.831559
H	-5.374613	-1.908664	-1.197873
C	-7.210911	-3.654506	0.669279
C	-6.530073	-4.665340	-0.044835
C	-5.145455	-4.882207	0.172737
C	-4.456626	-4.074999	1.108260
C	-5.123707	-3.037986	1.800338
C	-6.512122	-2.839929	1.586629
C	-4.461956	-5.971055	-0.620706
O	-5.145561	-6.784218	-1.343027
N	-3.093863	-6.036047	-0.533067
C	-2.303156	-7.034889	-1.266410
C	-1.088361	-6.403796	-1.942747
O	-1.585211	-5.551072	-3.044165
C	-0.617676	-4.874422	-3.803755

C	0.775187	-4.941825	-3.562921
C	1.676127	-4.258762	-4.413343
C	1.182221	-3.514755	-5.508683
C	-0.214560	-3.433562	-5.741391
C	-1.109820	-4.104237	-4.890335
O	1.998339	-2.824396	-6.430569
C	3.459662	-2.986206	-6.269522
C	4.137130	-2.219647	-7.387291
O	4.075914	-0.778674	-7.085219
C	4.660677	0.015382	-8.181358
C	4.830693	1.452225	-7.726748
O	3.508974	2.097784	-7.634734
C	3.635465	3.493567	-7.185471
C	2.242656	4.085935	-6.987313
O	1.416624	3.385861	-5.987974
C	2.075583	3.237997	-4.679135
C	0.947271	2.945643	-3.694414
O	1.585012	2.372678	-2.494667
C	0.718650	1.861274	-1.507900
C	-0.695550	1.944055	-1.575948
C	-1.477870	1.374528	-0.552406
C	-0.858867	0.727374	0.541577
C	0.556198	0.658604	0.616904
C	1.339760	1.226006	-0.408264
O	-1.725385	0.182427	1.507135
C	-1.096419	-0.579183	2.606137
C	-2.219416	-1.154092	3.468295

N	-3.036253	-2.113813	2.710474
C	-4.407192	-2.087742	2.727924
O	-5.070730	-1.261526	3.458749
H	-8.281360	-3.494933	0.503659
H	-7.052291	-5.299496	-0.768474
H	-3.396130	-4.260803	1.307023
H	-7.021514	-2.044691	2.140144
H	-1.935296	-7.816754	-0.571884
H	-2.960072	-7.520293	-2.006931
H	-0.513273	-5.787121	-1.224735
H	-0.434156	-7.198647	-2.352027
H	1.177527	-5.516815	-2.725850
H	2.747318	-4.327835	-4.208069
H	-0.584123	-2.856934	-6.595041
H	-2.189395	-4.053080	-5.062790
H	3.782055	-2.600017	-5.283986
H	3.720479	-4.060035	-6.341879
H	5.199217	-2.537878	-7.453514
H	3.644107	-2.434294	-8.358024
H	5.659859	-0.393573	-8.442630
H	4.011535	-0.041033	-9.079827
H	5.458108	1.993185	-8.467162
H	5.338848	1.486933	-6.740514
H	4.170771	4.095793	-7.951554
H	4.227188	3.533005	-6.247440
H	2.360369	5.152828	-6.699173
H	1.658014	4.028574	-7.920971

H	2.793227	2.394064	-4.695331
H	2.617765	4.165448	-4.404273
H	0.391190	3.864739	-3.428861
H	0.242389	2.220516	-4.139300
H	-1.197910	2.432546	-2.414340
H	-2.568430	1.423601	-0.603724
H	1.054199	0.173213	1.457401
H	2.431818	1.172045	-0.366618
H	-0.465777	0.092741	3.219128
H	-0.469468	-1.388756	2.182969
H	-2.881595	-0.355584	3.840029
H	-1.754002	-1.646851	4.345033
H	-2.545110	-5.346334	0.008803
H	-2.507900	-2.766000	2.104349
Cl	-0.895468	-4.060145	1.020972
Br	-2.190992	-2.280996	-1.101360
Br	1.496170	-2.705803	-0.607824

Table S4: Cartesian Coordenates of **3**.Cl^-

**3**.Cl^-	x	y	z
N	0.576150	-0.019496	-5.824316
C	1.659749	-0.311762	-5.042531
C	1.472556	-0.706211	-3.699749
C	0.161628	-0.760505	-3.176238
C	-0.943813	-0.458937	-4.004594
C	-0.704687	-0.096284	-5.344310
C	0.815109	0.316972	-7.258932

C	2.644347	-1.036340	-2.893977
N	3.927007	-0.642690	-3.284902
N	4.835858	-1.108252	-2.397836
N	4.113639	-1.818677	-1.416295
C	2.769327	-1.778067	-1.701370
C	4.850389	-2.437509	-0.299505
C	5.063128	-1.469580	0.864191
O	3.776968	-1.373717	1.576491
C	3.732972	-0.543517	2.716771
C	4.771113	0.331850	3.109418
C	4.615250	1.137497	4.264813
C	3.423771	1.062296	5.025648
C	2.388419	0.172526	4.631286
C	2.543158	-0.627386	3.485567
O	3.171872	1.814176	6.187593
C	4.186366	2.819812	6.569985
C	3.678437	3.539722	7.816941
O	2.462113	4.336478	7.602916
C	2.696198	5.597167	6.884578
C	1.376598	6.287998	6.646611
C	0.169765	5.784769	7.012195
C	-1.147905	6.463905	6.752610
O	-1.954300	5.531039	5.925211
C	-3.399692	5.810949	5.983699
C	-4.098755	4.727594	5.181065
O	-3.973460	5.069122	3.746318
C	-4.256443	4.056923	2.809563

C	-4.135427	4.436077	1.445804
C	-4.359970	3.491253	0.428216
C	-4.700985	2.156743	0.764899
C	-4.837337	1.775113	2.117585
C	-4.622656	2.729889	3.140974
O	-4.881935	1.279410	-0.331620
C	-4.989872	-0.155140	-0.008871
C	-5.241848	-0.900486	-1.321452
N	-4.193629	-0.651534	-2.326817
N	-4.469568	0.183694	-3.429020
N	-3.316379	0.288445	-4.130426
C	-2.313281	-0.453802	-3.498154
C	-2.880325	-1.049146	-2.355174
H	2.639580	-0.254563	-5.525628
H	0.000360	-1.018283	-2.129674
H	-1.511854	0.142766	-6.036540
H	1.035239	-0.619076	-7.797114
H	-0.087723	0.790682	-7.669273
H	1.673745	1.004191	-7.331716
H	5.827772	-2.748566	-0.700570
H	4.301439	-3.331379	0.036743
H	5.382917	-0.478221	0.491851
H	5.839795	-1.874422	1.541043
H	5.701189	0.405095	2.539255
H	5.430192	1.806840	4.552982
H	1.474773	0.115948	5.231181
H	1.756302	-1.322949	3.178998

H	5.144085	2.315299	6.810916
H	4.355515	3.517286	5.726970
H	4.498296	4.177960	8.210456
H	3.401962	2.800344	8.587900
H	3.380194	6.247342	7.476112
H	3.191045	5.411333	5.906017
H	1.443201	7.254007	6.124556
H	0.121200	4.815973	7.523623
H	-1.686802	6.653839	7.707396
H	-1.019104	7.430219	6.222538
H	-3.629630	6.817216	5.575497
H	-3.748842	5.769308	7.037784
H	-5.172317	4.666246	5.445356
H	-3.614922	3.755647	5.392657
H	-3.856034	5.465504	1.199856
H	-4.260147	3.771549	-0.625248
H	-5.083364	0.749472	2.410864
H	-4.739985	2.413327	4.180809
H	-5.854730	-0.339468	0.652373
H	-4.062549	-0.485593	0.493794
H	-6.186083	-0.568553	-1.779647
H	-5.305825	-1.982325	-1.117796
C	-7.187979	-3.691647	0.765530
C	-6.490597	-4.672876	0.025962
C	-5.092237	-4.835241	0.200069
C	-4.406718	-4.001857	1.114201
C	-5.093781	-3.001341	1.838867

C	-6.494863	-2.857176	1.669736
C	-4.391226	-5.908160	-0.600337
O	-5.062869	-6.721615	-1.334263
N	-3.023619	-5.967662	-0.495493
C	-2.225121	-6.974786	-1.208591
C	-1.011652	-6.350606	-1.893316
O	-1.514718	-5.515113	-3.006217
C	-0.554857	-4.841049	-3.777419
C	0.840229	-4.894790	-3.542846
C	1.730557	-4.212685	-4.405990
C	1.223658	-3.483528	-5.505523
C	-0.174806	-3.418414	-5.732761
C	-1.059570	-4.087078	-4.869953
O	2.026245	-2.788694	-6.434737
C	3.490690	-2.942016	-6.292335
C	4.147702	-2.159564	-7.411184
O	4.079922	-0.721523	-7.094941
C	4.654168	0.084064	-8.188529
C	4.803911	1.521850	-7.730278
O	3.472866	2.147782	-7.636919
C	3.579840	3.549073	-7.199730
C	2.178934	4.119855	-6.996103
O	1.370197	3.410619	-5.989196
C	2.041871	3.270359	-4.685961
C	0.924224	2.955236	-3.696715
O	1.574207	2.391793	-2.498516
C	0.714210	1.879540	-1.506152

C	-0.701215	1.923930	-1.587320
C	-1.477164	1.357014	-0.558327
C	-0.852365	0.754158	0.557427
C	0.563796	0.724639	0.647239
C	1.341537	1.285910	-0.386930
O	-1.716100	0.217976	1.528485
C	-1.084605	-0.518762	2.643255
C	-2.208341	-1.090075	3.506391
N	-3.015097	-2.058717	2.749888
C	-4.385308	-2.040119	2.761741
O	-5.057982	-1.217313	3.489697
H	-8.268483	-3.573636	0.633182
H	-7.010305	-5.325814	-0.682697
H	-3.329174	-4.131632	1.263521
H	-7.018171	-2.087902	2.246682
H	-1.854894	-7.741538	-0.498590
H	-2.876497	-7.477181	-1.942505
H	-0.437919	-5.722983	-1.183828
H	-0.355579	-7.148137	-2.293569
H	1.251691	-5.459853	-2.703120
H	2.803902	-4.273186	-4.208893
H	-0.553578	-2.853770	-6.590313
H	-2.140340	-4.049566	-5.038892
H	3.823568	-2.562953	-5.307736
H	3.757029	-4.013434	-6.378014
H	5.211008	-2.469479	-7.494380
H	3.642993	-2.367811	-8.377284

H	5.658780	-0.310849	-8.450418
H	4.006172	0.021355	-9.087387
H	5.423402	2.073133	-8.469693
H	5.311562	1.562061	-6.743995
H	4.099420	4.153601	-7.974706
H	4.178013	3.605857	-6.266709
H	2.280954	5.189763	-6.713309
H	1.589804	4.048756	-7.925983
H	2.774120	2.439439	-4.710755
H	2.569199	4.206368	-4.411422
H	0.351958	3.863269	-3.427667
H	0.231460	2.217759	-4.139820
H	-1.209624	2.379883	-2.440102
H	-2.568124	1.366513	-0.623357
H	1.069021	0.282725	1.508224
H	2.434557	1.265450	-0.331940
H	-0.462769	0.168618	3.247686
H	-0.450541	-1.329302	2.232763
H	-2.875069	-0.291230	3.869334
H	-1.744351	-1.573681	4.389082
H	-2.479436	-5.284675	0.065365
H	-2.474183	-2.706000	2.143336
Cl	-0.897775	-3.979477	1.098118
Cl	-2.190237	-2.158277	-1.140031
Cl	1.549173	-2.609313	-0.712934

Table S5: Cartesian Coordenates of **4**.Cl^-

**4**.Cl^-	x	y	z
N	0.633163	0.137925	-5.789759
C	1.730659	-0.212422	-5.053983
C	1.569482	-0.712760	-3.744445
C	0.269778	-0.828410	-3.200102
C	-0.848146	-0.458399	-3.982292
C	-0.636293	0.018952	-5.290600
C	0.837624	0.590461	-7.198068
C	2.753070	-1.103145	-2.989445
N	4.050771	-0.810715	-3.416348
N	4.949629	-1.339450	-2.550579
N	4.204474	-1.991612	-1.541948
C	2.870533	-1.841208	-1.800902
C	4.904438	-2.658684	-0.428570
C	5.086886	-1.736418	0.776684
O	3.784638	-1.678074	1.464752
C	3.695879	-0.857507	2.611014
C	4.697661	0.049974	3.024265
C	4.487394	0.858637	4.168768
C	3.277508	0.756346	4.896730
C	2.285240	-0.175701	4.490111
C	2.494847	-0.980655	3.356510
O	2.967345	1.522482	6.035225
C	3.954719	2.545289	6.444098
C	3.374850	3.302606	7.636196
O	2.200309	4.128155	7.317848

C	2.527565	5.360694	6.587147
C	1.264073	6.141609	6.326300
C	0.020810	5.744036	6.699534
C	-1.237245	6.521312	6.418708
O	-2.116634	5.636246	5.614564
C	-3.533809	6.038350	5.650384
C	-4.317023	4.965928	4.912567
O	-4.166079	5.204400	3.458929
C	-4.412567	4.126395	2.587450
C	-4.233663	4.405184	1.205814
C	-4.405186	3.385626	0.251408
C	-4.750468	2.075353	0.670347
C	-4.954185	1.796379	2.038996
C	-4.793179	2.825752	2.997681
O	-4.873003	1.114856	-0.362827
C	-4.876092	-0.302215	0.048948
C	-5.066466	-1.136228	-1.220424
N	-4.060451	-0.835719	-2.254491
N	-4.417854	-0.079286	-3.391944
N	-3.283952	0.096416	-4.112876
C	-2.209798	-0.523029	-3.465887
C	-2.722747	-1.106891	-2.297233
H	2.700700	-0.121500	-5.549576
H	0.127624	-1.201606	-2.184487
H	-1.458974	0.312339	-5.942449
H	0.980439	-0.303542	-7.826229
H	-0.051731	1.147542	-7.525161

H	1.730265	1.234515	-7.246737
H	5.890152	-2.958751	-0.817123
H	4.340453	-3.561995	-0.144976
H	5.407048	-0.729913	0.448327
H	5.851381	-2.161881	1.454336
H	5.639938	0.148553	2.478483
H	5.274423	1.553820	4.472604
H	1.360243	-0.259417	5.068948
H	1.740175	-1.706967	3.039507
H	4.898275	2.050483	6.751326
H	4.169439	3.219445	5.592788
H	4.179588	3.929907	8.076017
H	3.023225	2.587319	8.399090
H	3.249638	5.969071	7.178265
H	3.018136	5.128488	5.616325
H	1.405923	7.085416	5.779165
H	-0.105797	4.795311	7.234864
H	-1.759360	6.779663	7.366818
H	-1.028809	7.460117	5.864794
H	-3.679895	7.033670	5.181390
H	-3.885057	6.089238	6.703426
H	-5.392273	5.003729	5.172658
H	-3.907664	3.977201	5.190041
H	-3.946847	5.415575	0.897364
H	-4.256990	3.588831	-0.814053
H	-5.213294	0.794762	2.397317
H	-4.962932	2.585306	4.050443

H	-5.728630	-0.518021	0.717699
H	-3.928357	-0.530764	0.571022
H	-6.042651	-0.923796	-1.682752
H	-5.016648	-2.206496	-0.962534
C	-7.176855	-4.103123	1.506263
C	-6.441845	-4.930235	0.626858
C	-5.035703	-4.779632	0.517502
C	-4.383213	-3.782690	1.279340
C	-5.109711	-2.971282	2.180888
C	-6.514571	-3.131636	2.289536
C	-4.280177	-5.710851	-0.400324
O	-4.904623	-6.462835	-1.234496
N	-2.913850	-5.729681	-0.260027
C	-2.057329	-6.628725	-1.043606
C	-0.896850	-5.896329	-1.714316
O	-1.462025	-5.057616	-2.797593
C	-0.544328	-4.470826	-3.683592
C	0.861615	-4.517993	-3.520919
C	1.706812	-3.941682	-4.498087
C	1.145484	-3.304924	-5.628386
C	-0.263184	-3.254772	-5.789156
C	-1.103369	-3.830922	-4.821374
O	1.900674	-2.674648	-6.639158
C	3.370085	-2.840436	-6.569301
C	3.980041	-2.037083	-7.700366
O	4.000668	-0.616252	-7.307333
C	4.537723	0.228651	-8.389310

C	4.838096	1.610382	-7.839909
O	3.572321	2.321828	-7.587833
C	3.823099	3.646375	-6.996564
C	2.488293	4.326875	-6.705767
O	1.615895	3.587944	-5.777180
C	2.258266	3.279009	-4.488786
C	1.109044	2.928693	-3.548845
O	1.713660	2.236573	-2.395484
C	0.817259	1.729654	-1.433687
C	-0.592792	1.852381	-1.525201
C	-1.408038	1.309189	-0.513511
C	-0.827075	0.650327	0.594879
C	0.583734	0.519621	0.681764
C	1.400157	1.060835	-0.333117
O	-1.724944	0.168970	1.563646
C	-1.131758	-0.426629	2.782096
C	-2.285410	-0.855862	3.686141
N	-3.070364	-1.928830	3.057473
C	-4.439115	-1.904867	3.008529
O	-5.139036	-1.012718	3.620180
H	-8.262214	-4.223130	1.588336
H	-6.937898	-5.696383	0.022162
H	-3.304829	-3.625947	1.159287
H	-7.067356	-2.487817	2.981301
H	-1.622845	-7.403818	-0.380915
H	-2.683594	-7.134567	-1.796605
H	-0.354088	-5.257387	-0.990349

H	-0.199227	-6.639091	-2.147943
H	1.315616	-5.004464	-2.654045
H	2.788592	-3.998572	-4.355189
H	-0.685981	-2.769375	-6.674106
H	-2.191099	-3.807749	-4.940326
H	3.756968	-2.477098	-5.598622
H	3.619369	-3.913303	-6.681803
H	5.019700	-2.385207	-7.877119
H	3.397514	-2.175328	-8.634447
H	5.481975	-0.213229	-8.772388
H	3.810890	0.285853	-9.226029
H	5.439578	2.175091	-8.584023
H	5.422867	1.530012	-6.899718
H	4.395364	4.281729	-7.707264
H	4.431688	3.533932	-6.075123
H	2.698664	5.340170	-6.300757
H	1.892265	4.425960	-7.628678
H	2.944367	2.415082	-4.588737
H	2.831603	4.151846	-4.115781
H	0.571882	3.834538	-3.209513
H	0.393391	2.264065	-4.065277
H	-1.065424	2.362290	-2.367951
H	-2.495871	1.394884	-0.579205
H	1.055977	0.015716	1.527131
H	2.489723	0.974379	-0.273948
H	-0.506746	0.327138	3.297058
H	-0.512974	-1.300506	2.500999

H	-2.959557	-0.010848	3.900053
H	-1.851838	-1.199662	4.646231
H	-2.415890	-5.134822	0.434936
H	-2.510865	-2.676232	2.594878
Cl	-0.917241	-3.925303	1.596180
F	-2.112271	-1.856055	-1.322363
F	1.910159	-2.402973	-0.999471

Table S6: Cartesian Coordenates of **5**.Cl^-

**5**.Cl^-	x	y	z
N	0.587420	-0.044035	-5.900583
C	1.629113	-0.375779	-5.080221
C	1.400345	-0.685962	-3.720059
C	0.073495	-0.625860	-3.227110
C	-0.991194	-0.272280	-4.094282
C	-0.703558	0.008323	-5.441559
C	0.880737	0.214699	-7.340020
C	2.563853	-0.982671	-2.884124
N	3.823421	-0.516010	-3.285752
N	4.745481	-0.896179	-2.381875
N	4.048504	-1.614547	-1.381410
C	2.692576	-1.698073	-1.658685
C	4.836264	-2.053343	-0.215948
C	5.020281	-0.944668	0.821591
O	3.764254	-0.853353	1.590898
C	3.759918	0.006075	2.709081
C	4.767729	0.957428	2.987829

C	4.674157	1.767103	4.147123
C	3.575127	1.618871	5.027122
C	2.557931	0.670508	4.734341
C	2.650431	-0.132039	3.583949
O	3.404923	2.344839	6.220554
C	4.393261	3.405880	6.509557
C	4.037713	4.015221	7.863894
O	2.730524	4.684968	7.899905
C	2.713131	5.998270	7.240727
C	1.287183	6.476231	7.123988
C	0.204151	5.754979	7.512247
C	-1.219368	6.218252	7.361854
O	-1.898900	5.231915	6.484206
C	-3.365571	5.356845	6.521155
C	-3.932721	4.318243	5.570388
O	-3.765608	4.829711	4.192194
C	-4.068538	3.947218	3.137617
C	-3.919001	4.482120	1.830303
C	-4.184018	3.679332	0.706406
C	-4.595165	2.332744	0.879266
C	-4.745964	1.792752	2.174569
C	-4.486798	2.604883	3.305897
O	-4.830074	1.617291	-0.315809
C	-5.110277	0.174032	-0.200288
C	-5.418774	-0.318908	-1.616411
N	-4.287977	-0.114115	-2.540025
N	-4.336538	0.957871	-3.450413

N	-3.146833	0.961285	-4.098240
C	-2.360260	-0.088814	-3.608642
C	-3.081586	-0.788929	-2.614597
H	2.620979	-0.395230	-5.540275
H	-0.131744	-0.777576	-2.167005
H	-1.475923	0.288563	-6.157816
H	1.059491	-0.752758	-7.836128
H	0.018716	0.721726	-7.796695
H	1.779115	0.848988	-7.414592
H	5.826843	-2.355853	-0.592546
H	4.353518	-2.925342	0.246075
H	5.251093	0.019575	0.330661
H	5.851404	-1.215154	1.501423
H	5.627503	1.084004	2.324210
H	5.465570	2.494722	4.345736
H	1.710511	0.567013	5.419331
H	1.881919	-0.878360	3.359283
H	5.410514	2.969113	6.572927
H	4.379597	4.156649	5.695621
H	4.847360	4.718367	8.153567
H	3.969444	3.218736	8.624449
H	3.325934	6.722050	7.824044
H	3.160885	5.939456	6.224465
H	1.162392	7.471377	6.672279
H	0.348496	4.761407	7.953069
H	-1.736100	6.238147	8.347282
H	-1.279172	7.231640	6.912808

H	-3.685609	6.377333	6.223405
H	-3.737340	5.156874	7.549233
H	-5.009055	4.148788	5.769956
H	-3.384693	3.364650	5.695978
H	-3.592558	5.520121	1.710710
H	-4.067297	4.078947	-0.306060
H	-5.034443	0.749404	2.340705
H	-4.614851	2.170524	4.301082
H	-5.998964	-0.001219	0.434001
H	-4.234848	-0.341658	0.236778
H	-6.272090	0.236643	-2.035728
H	-5.673567	-1.390116	-1.580584
C	-7.226905	-3.927875	0.618671
C	-6.536323	-5.005492	0.021805
C	-5.146336	-5.174063	0.248205
C	-4.465756	-4.261702	1.087337
C	-5.146994	-3.168813	1.672814
C	-6.535375	-3.006434	1.436395
C	-4.439813	-6.296606	-0.472738
O	-5.103136	-7.171711	-1.138530
N	-3.067192	-6.307662	-0.411198
C	-2.257181	-7.301469	-1.130596
C	-1.055459	-6.655401	-1.817015
O	-1.569413	-5.792849	-2.904976
C	-0.612262	-5.099438	-3.669419
C	0.781731	-5.167620	-3.447262
C	1.669050	-4.445832	-4.280943

C	1.158503	-3.662274	-5.340274
C	-0.242138	-3.577028	-5.550064
C	-1.122518	-4.289715	-4.718081
O	1.961364	-2.945735	-6.252220
C	3.423781	-3.136054	-6.131452
C	4.089304	-2.413669	-7.285367
O	4.076792	-0.964894	-7.021077
C	4.700928	-0.219452	-8.129416
C	4.911225	1.222120	-7.707353
O	3.608768	1.908369	-7.646720
C	3.767216	3.307303	-7.217598
C	2.389002	3.942808	-7.052870
O	1.535594	3.302010	-6.037375
C	2.168266	3.216003	-4.709635
C	1.017842	3.009408	-3.728621
O	1.626121	2.513515	-2.478677
C	0.743329	1.975294	-1.521820
C	-0.669022	2.060404	-1.614175
C	-1.472451	1.438960	-0.638150
C	-0.873490	0.740282	0.435391
C	0.541012	0.689644	0.550300
C	1.343918	1.307996	-0.430288
O	-1.758881	0.129989	1.344743
C	-1.146677	-0.613677	2.465890
C	-2.280110	-1.198722	3.306767
N	-3.075763	-2.180146	2.555109
C	-4.446706	-2.138028	2.520076

O	-5.124420	-1.246619	3.155398
H	-8.299460	-3.800264	0.439147
H	-7.052226	-5.719701	-0.627791
H	-3.399831	-4.404674	1.291954
H	-7.052017	-2.160149	1.900309
H	-1.871687	-8.068313	-0.428897
H	-2.905371	-7.810197	-1.863160
H	-0.476075	-6.040179	-1.101232
H	-0.401501	-7.441858	-2.241263
H	1.196941	-5.775153	-2.640445
H	2.742642	-4.510452	-4.087318
H	-0.627050	-2.965964	-6.372284
H	-2.203487	-4.236923	-4.875990
H	3.780876	-2.738893	-5.162229
H	3.658454	-4.216637	-6.190811
H	5.140409	-2.762971	-7.369878
H	3.565963	-2.640095	-8.237449
H	5.689470	-0.667350	-8.366436
H	4.062222	-0.277490	-9.035277
H	5.563110	1.726019	-8.452664
H	5.408941	1.264541	-6.716195
H	4.332474	3.880337	-7.984595
H	4.343976	3.345275	-6.270278
H	2.533211	5.015707	-6.801020
H	1.811899	3.868602	-7.990057
H	2.864427	2.355551	-4.662523
H	2.729353	4.145102	-4.481778

H	0.470010	3.951839	-3.538929
H	0.312338	2.262190	-4.133374
H	-1.153426	2.588512	-2.439172
H	-2.562321	1.487007	-0.709826
H	1.026981	0.173121	1.379235
H	2.434702	1.255894	-0.364574
H	-0.544961	0.074686	3.090318
H	-0.490581	-1.412143	2.066469
H	-2.959039	-0.404472	3.655031
H	-1.824255	-1.675542	4.197231
H	-2.533074	-5.603791	0.128663
H	-2.536305	-2.899641	2.041618
Cl	-0.914416	-4.343173	1.180933
Te	-2.640195	-2.575805	-1.573337
Te	1.192019	-2.692902	-0.489844
C	2.347688	-4.526963	-0.262849
H	2.836509	-4.553675	0.723078
H	1.630995	-5.357999	-0.345756
H	3.092208	-4.583741	-1.072505
C	-4.180639	-3.660970	-2.674272
H	-4.313878	-3.163486	-3.648292
H	-3.792545	-4.679955	-2.820462
H	-5.125467	-3.673437	-2.112878

Table S7: Cartesian Coordenates of **6**.Cl^-

**6**.Cl^-	x	y	z
N	0.687740000	0.035940000	-5.919511000

C	1.749460000	-0.246458000	-5.107694000
C	1.539490000	-0.551252000	-3.743959000
C	0.217285000	-0.556102000	-3.242342000
C	-0.869877000	-0.259859000	-4.102807000
C	-0.600093000	0.030504000	-5.453340000
C	0.954900000	0.300814000	-7.362238000
C	2.722320000	-0.787354000	-2.914343000
N	3.954187000	-0.252373000	-3.312111000
N	4.900666000	-0.583498000	-2.409557000
N	4.254293000	-1.350177000	-1.416325000
C	2.909086000	-1.489806000	-1.698082000
I	1.555831000	-2.610849000	-0.536431000
C	5.061768000	-1.829843000	-0.281068000
C	5.141260000	-0.813683000	0.858681000
O	3.851212000	-0.861453000	1.571033000
C	3.743592000	-0.093979000	2.751159000
C	4.678430000	0.888194000	3.148866000
C	4.478139000	1.605114000	4.353228000
C	3.346601000	1.333175000	5.158513000
C	2.407258000	0.347553000	4.753021000
C	2.606116000	-0.363983000	3.555742000
O	3.075113000	1.974415000	6.382281000
C	4.029583000	3.022503000	6.807113000
C	3.562536000	3.555112000	8.158185000
O	2.281842000	4.278139000	8.109065000
C	2.391578000	5.630893000	7.546264000
C	1.011476000	6.207483000	7.353372000

C	-0.142833000	5.538783000	7.606467000
C	-1.518033000	6.109048000	7.388301000
O	-2.203404000	5.217957000	6.418184000
C	-3.663316000	5.407087000	6.402423000
C	-4.241580000	4.384758000	5.440620000
O	-4.024002000	4.877949000	4.062197000
C	-4.290471000	3.979902000	3.010764000
C	-4.097304000	4.493279000	1.700247000
C	-4.311688000	3.668906000	0.580668000
C	-4.716005000	2.321490000	0.760639000
C	-4.916987000	1.806326000	2.059649000
C	-4.709194000	2.638843000	3.185826000
O	-4.886876000	1.571878000	-0.427296000
C	-5.110454000	0.124228000	-0.261935000
C	-5.326807000	-0.465595000	-1.656709000
N	-4.192416000	-0.237193000	-2.568647000
N	-4.325468000	0.711428000	-3.604607000
N	-3.135277000	0.742312000	-4.246401000
C	-2.253669000	-0.157853000	-3.634140000
C	-2.929121000	-0.788560000	-2.565177000
I	-2.290333000	-2.263713000	-1.211756000
H	2.737145000	-0.243240000	-5.577606000
H	0.035471000	-0.745805000	-2.184834000
H	-1.388816000	0.266889000	-6.167407000
H	1.083251000	-0.665471000	-7.876191000
H	0.101236000	0.845518000	-7.790392000
H	1.872977000	0.903968000	-7.447358000

H	6.076338000	-2.013539000	-0.669149000
H	4.638697000	-2.780703000	0.080329000
H	5.337304000	0.201691000	0.466074000
H	5.959528000	-1.099943000	1.546861000
H	5.564349000	1.114400000	2.550350000
H	5.210503000	2.361557000	4.641870000
H	1.537771000	0.143741000	5.385947000
H	1.898949000	-1.138177000	3.241678000
H	5.041091000	2.584323000	6.919667000
H	4.072484000	3.819638000	6.040782000
H	4.360703000	4.205918000	8.573845000
H	3.389773000	2.715264000	8.852599000
H	2.995231000	6.274696000	8.225319000
H	2.917743000	5.612512000	6.566883000
H	0.986145000	7.235694000	6.963037000
H	-0.098921000	4.510964000	7.986084000
H	-2.097966000	6.116045000	8.338140000
H	-1.478544000	7.145511000	6.993170000
H	-3.929124000	6.438698000	6.090153000
H	-4.078622000	5.228338000	7.417740000
H	-5.327851000	4.252226000	5.611552000
H	-3.728318000	3.415631000	5.588572000
H	-3.771936000	5.530879000	1.574551000
H	-4.155842000	4.052412000	-0.432744000
H	-5.200332000	0.764505000	2.236534000
H	-4.873169000	2.218364000	4.181417000
H	-6.024473000	-0.061768000	0.331012000

H	-4.241406000	-0.324287000	0.253462000
H	-6.204506000	-0.005484000	-2.136746000
H	-5.498919000	-1.551006000	-1.560177000
C	-7.080817000	-3.569844000	0.395640000
C	-6.396792000	-4.667050000	-0.172584000
C	-5.056905000	-4.943401000	0.201274000
C	-4.417146000	-4.113656000	1.152805000
C	-5.083901000	-2.990393000	1.695629000
C	-6.427329000	-2.729941000	1.323204000
C	-4.361224000	-6.105152000	-0.467056000
O	-5.026297000	-6.973177000	-1.140500000
N	-2.995182000	-6.161607000	-0.338127000
C	-2.182830000	-7.189238000	-1.005819000
C	-0.967613000	-6.575880000	-1.700454000
O	-1.475625000	-5.702291000	-2.780284000
C	-0.521483000	-5.004374000	-3.539549000
C	0.876340000	-5.094212000	-3.344184000
C	1.757701000	-4.375848000	-4.188095000
C	1.239119000	-3.579877000	-5.234604000
C	-0.163928000	-3.471817000	-5.415393000
C	-1.039007000	-4.173742000	-4.569098000
O	2.033364000	-2.869863000	-6.159832000
C	3.494376000	-3.084457000	-6.076156000
C	4.143685000	-2.371651000	-7.245739000
O	4.160628000	-0.924360000	-6.977865000
C	4.800932000	-0.179933000	-8.076373000
C	5.013548000	1.257124000	-7.639124000

O	3.711071000	1.942838000	-7.575352000
C	3.860321000	3.325661000	-7.093863000
C	2.479237000	3.957317000	-6.936632000
O	1.611984000	3.300215000	-5.943801000
C	2.215206000	3.228907000	-4.601361000
C	1.049747000	3.018680000	-3.638565000
O	1.646636000	2.522003000	-2.385027000
C	0.760444000	2.017224000	-1.413358000
C	-0.652688000	2.063100000	-1.526717000
C	-1.452919000	1.488315000	-0.519045000
C	-0.851898000	0.876577000	0.605595000
C	0.561255000	0.857779000	0.732749000
C	1.362125000	1.426770000	-0.278490000
O	-1.733095000	0.315739000	1.549598000
C	-1.121747000	-0.464063000	2.645062000
C	-2.256967000	-1.080628000	3.461802000
N	-3.041367000	-2.038709000	2.668204000
C	-4.412566000	-2.019045000	2.633393000
O	-5.109572000	-1.188634000	3.326098000
H	-8.116572000	-3.362689000	0.107501000
H	-6.882132000	-5.319747000	-0.905055000
H	-3.400672000	-4.348747000	1.482664000
H	-6.938928000	-1.870218000	1.767364000
H	-1.814071000	-7.931697000	-0.270298000
H	-2.826003000	-7.717544000	-1.728931000
H	-0.364940000	-5.976303000	-0.990958000
H	-0.337820000	-7.379246000	-2.129796000

H	1.298499000	-5.710132000	-2.546468000
H	2.833695000	-4.453837000	-4.012447000
H	-0.555400000	-2.851388000	-6.227116000
H	-2.122800000	-4.100400000	-4.701906000
H	3.883777000	-2.694941000	-5.116377000
H	3.709509000	-4.168689000	-6.142025000
H	5.186534000	-2.737988000	-7.355092000
H	3.594214000	-2.587395000	-8.185345000
H	5.788863000	-0.632743000	-8.305852000
H	4.170937000	-0.227847000	-8.988832000
H	5.668987000	1.768649000	-8.375811000
H	5.506968000	1.287015000	-6.645607000
H	4.441910000	3.924106000	-7.828424000
H	4.416556000	3.329609000	-6.133830000
H	2.619505000	5.025546000	-6.663997000
H	1.916039000	3.900116000	-7.883386000
H	2.917750000	2.375536000	-4.532698000
H	2.763712000	4.164176000	-4.369344000
H	0.497827000	3.959267000	-3.451505000
H	0.349328000	2.273190000	-4.055122000
H	-1.139349000	2.525484000	-2.389131000
H	-2.542149000	1.498756000	-0.610402000
H	1.046826000	0.409581000	1.601568000
H	2.452983000	1.405719000	-0.197596000
H	-0.520523000	0.200003000	3.294838000
H	-0.464228000	-1.247507000	2.218745000
H	-2.942905000	-0.301803000	3.831904000

H	-1.805593000	-1.584052000	4.339582000
H	-2.466495000	-5.422477000	0.154639000
H	-2.496546000	-2.689911000	2.078414000
F	7.370240000	2.685081000	2.835750000
F	9.312163000	3.596670000	5.615003000
F	8.747162000	4.653569000	3.438234000
F	7.929288000	1.627887000	5.013011000
F	6.938956000	3.902513000	4.959256000
F	9.741738000	2.376689000	3.491887000
P	8.345900000	3.140070000	4.225745000
Cl	-0.853118000	-4.021722000	1.029438000

Table S8: Cartesian Coordenates of **1**.Br^-

**1**.Br^-	x	y	z
N	0.633424	-0.044824	-5.935750
C	1.715841	-0.345595	-5.158455
C	1.541822	-0.672737	-3.794921
C	0.233951	-0.681932	-3.256784
C	-0.875111	-0.370355	-4.082577
C	-0.640547	-0.056647	-5.435113
C	0.860871	0.239159	-7.381741
C	2.746387	-0.922120	-3.000339
N	3.967942	-0.384385	-3.426597
N	4.938155	-0.721411	-2.551737
N	4.318126	-1.495682	-1.547931
C	2.965639	-1.633900	-1.794888
I	1.653988	-2.776006	-0.596122

C	5.152945	-1.965672	-0.429116
C	5.272265	-0.923557	0.683963
O	4.011295	-0.958709	1.448353
C	3.927693	-0.127677	2.585174
C	4.855667	0.892128	2.898009
C	4.677389	1.676030	4.064826
C	3.573438	1.435265	4.917959
C	2.643587	0.409292	4.598147
C	2.821036	-0.369319	3.440819
O	3.316562	2.142448	6.106861
C	4.242065	3.245491	6.445157
C	3.782537	3.844972	7.771830
O	2.482847	4.529030	7.704086
C	2.550687	5.858306	7.080573
C	1.157379	6.417384	6.938623
C	0.024872	5.761247	7.299872
C	-1.364080	6.314252	7.128238
O	-2.090223	5.374193	6.237855
C	-3.549736	5.568057	6.270557
C	-4.162755	4.513141	5.367068
O	-3.989048	4.956657	3.965756
C	-4.251671	4.013159	2.954209
C	-4.087962	4.480628	1.622671
C	-4.298608	3.609856	0.538213
C	-4.669556	2.261564	0.774817
C	-4.842122	1.791617	2.095101
C	-4.639277	2.671197	3.185903

O	-4.840398	1.465425	-0.381838
C	-5.033465	0.020004	-0.164986
C	-5.273571	-0.614335	-1.536258
N	-4.164793	-0.383738	-2.478271
N	-4.330228	0.560658	-3.513345
N	-3.152985	0.606805	-4.177219
C	-2.247474	-0.280374	-3.580834
C	-2.894000	-0.917278	-2.498009
I	-2.214152	-2.394072	-1.162765
H	2.690020	-0.341158	-5.655893
H	0.081520	-0.887116	-2.197349
H	-1.447823	0.194012	-6.123096
H	0.966731	-0.721740	-7.910672
H	-0.001135	0.795802	-7.776630
H	1.781665	0.835082	-7.485905
H	6.154192	-2.167763	-0.841892
H	4.731513	-2.904651	-0.036721
H	5.444123	0.082052	0.255759
H	6.117939	-1.187617	1.347602
H	5.717326	1.092475	2.255375
H	5.407293	2.458157	4.289693
H	1.796348	0.229028	5.267075
H	2.119840	-1.173168	3.195604
H	5.270325	2.848284	6.565370
H	4.243152	3.995101	5.630045
H	4.569230	4.540479	8.133739
H	3.646651	3.042269	8.516623

H	3.184061	6.533437	7.699693
H	3.024182	5.801310	6.075778
H	1.101499	7.421691	6.493287
H	0.097354	4.757446	7.735296
H	-1.893794	6.364062	8.105657
H	-1.352445	7.330768	6.682442
H	-3.824233	6.588492	5.930492
H	-3.927279	5.427801	7.306383
H	-5.242982	4.389958	5.578019
H	-3.646354	3.548748	5.533657
H	-3.789154	5.520061	1.453438
H	-4.167054	3.958337	-0.491180
H	-5.104338	0.751675	2.312140
H	-4.783522	2.286266	4.198875
H	-5.928606	-0.162823	0.456582
H	-4.142102	-0.397002	0.339377
H	-6.172208	-0.185630	-2.006477
H	-5.419196	-1.700023	-1.407509
C	-7.236027	-3.497221	0.548261
C	-6.595619	-4.565155	-0.116758
C	-5.237295	-4.867534	0.158002
C	-4.532180	-4.085597	1.105171
C	-5.156167	-2.988957	1.746181
C	-6.519533	-2.707700	1.472599
C	-4.606134	-6.000857	-0.617458
O	-5.320861	-6.757394	-1.368578
N	-3.247069	-6.161849	-0.498735

C	-2.503340	-7.182918	-1.252160
C	-1.275658	-6.594139	-1.946646
O	-1.759428	-5.674816	-2.999220
C	-0.783447	-4.996019	-3.748301
C	0.610172	-5.107959	-3.533038
C	1.515304	-4.411642	-4.370287
C	1.025065	-3.615248	-5.429997
C	-0.373469	-3.483985	-5.630334
C	-1.272222	-4.163990	-4.790806
O	1.844307	-2.924628	-6.348452
C	3.297830	-3.188054	-6.265457
C	3.971426	-2.472421	-7.419991
O	4.043974	-1.033972	-7.113482
C	4.678038	-0.279036	-8.208637
C	4.929461	1.143272	-7.744489
O	3.641962	1.851337	-7.632690
C	3.830694	3.223735	-7.135483
C	2.466582	3.880558	-6.938397
O	1.605690	3.220814	-5.941494
C	2.230715	3.113391	-4.611494
C	1.076486	2.900860	-3.635595
O	1.681453	2.371189	-2.398795
C	0.797116	1.873941	-1.421311
C	-0.616065	1.952665	-1.515968
C	-1.416984	1.387745	-0.503688
C	-0.817232	0.751592	0.608142
C	0.596546	0.696956	0.715498

C	1.398536	1.256431	-0.300813
O	-1.700504	0.206149	1.560544
C	-1.093977	-0.595984	2.642929
C	-2.229812	-1.188948	3.477885
N	-3.058078	-2.122324	2.698789
C	-4.428551	-2.054865	2.681762
O	-5.081171	-1.202088	3.389727
H	-8.286616	-3.272832	0.337556
H	-7.128454	-5.179066	-0.849797
H	-3.497206	-4.340215	1.357916
H	-6.995720	-1.868603	1.989556
H	-2.154797	-7.984547	-0.571165
H	-3.192737	-7.633107	-1.985063
H	-0.642246	-6.038990	-1.227508
H	-0.680010	-7.407740	-2.405209
H	1.011660	-5.727814	-2.727780
H	2.587502	-4.510682	-4.182219
H	-0.742625	-2.864772	-6.453485
H	-2.352601	-4.074422	-4.940838
H	3.699604	-2.828557	-5.298801
H	3.476835	-4.277642	-6.349851
H	4.999120	-2.874960	-7.544307
H	3.410074	-2.641718	-8.362003
H	5.651067	-0.747415	-8.468832
H	4.028364	-0.292889	-9.108422
H	5.576195	1.658808	-8.486190
H	5.447878	1.142022	-6.763162

H	4.407828	3.821112	-7.874557
H	4.408290	3.202522	-6.188109
H	2.634090	4.940171	-6.647774
H	1.883239	3.854105	-7.874315
H	2.921932	2.248813	-4.571440
H	2.796435	4.036204	-4.370108
H	0.541099	3.845786	-3.423475
H	0.359740	2.173679	-4.056952
H	-1.103022	2.433417	-2.368055
H	-2.506581	1.425250	-0.582018
H	1.083059	0.230321	1.573803
H	2.489762	1.206507	-0.234670
H	-0.467609	0.049537	3.288401
H	-0.461270	-1.393017	2.203543
H	-2.887813	-0.394874	3.865571
H	-1.774160	-1.708389	4.343972
H	-2.676225	-5.533858	0.086510
H	-2.544209	-2.804758	2.120881
Br	-0.743783	-4.323854	1.155429

Table S9: Cartesian Coordenates of **1**.I^-

1.I^-	x	y	z
N	0.520423	-0.147717	-5.722905
C	1.672044	-0.253425	-4.994735
C	1.615461	-0.565530	-3.618917
C	0.351512	-0.768290	-3.016096
C	-0.830077	-0.651587	-3.789574

C	-0.710412	-0.341809	-5.159070
C	0.640097	0.117577	-7.184976
C	2.879051	-0.592749	-2.878714
N	3.967919	0.148049	-3.357213
N	5.015164	0.001395	-2.518789
N	4.582231	-0.857686	-1.485856
C	3.268620	-1.238158	-1.679854
I	2.234780	-2.588469	-0.430271
C	5.527741	-1.155945	-0.396183
C	5.513942	-0.083821	0.694116
O	4.291763	-0.290790	1.493933
C	4.100037	0.559501	2.603928
C	4.870460	1.715350	2.867500
C	4.588775	2.507871	4.008111
C	3.539151	2.140669	4.884155
C	2.769686	0.976609	4.615817
C	3.050681	0.189229	3.485615
O	3.188265	2.848992	6.048166
C	3.956805	4.077495	6.344004
C	3.424107	4.654175	7.653377
O	2.046861	5.162169	7.571891
C	1.942328	6.474087	6.916670
C	0.488546	6.851455	6.781441
C	-0.547299	6.076546	7.193887
C	-1.997784	6.444323	7.034009
O	-2.624034	5.366445	6.228381
C	-4.095011	5.380474	6.297791

C	-4.592594	4.194539	5.490599
O	-4.519292	4.556997	4.057496
C	-4.658122	3.517672	3.118336
C	-4.597954	3.913498	1.755256
C	-4.694339	2.949270	0.735848
C	-4.845685	1.578854	1.069068
C	-4.916482	1.178514	2.421384
C	-4.830156	2.151315	3.447059
O	-4.912925	0.691141	-0.029985
C	-4.857404	-0.748194	0.283279
C	-5.020986	-1.509658	-1.033643
N	-4.004470	-1.144569	-2.034647
N	-4.368349	-0.292550	-3.098556
N	-3.241992	-0.076246	-3.814527
C	-2.175330	-0.767027	-3.224576
C	-2.661758	-1.452264	-2.088607
I	-1.703659	-2.750384	-0.739479
H	2.608001	-0.119392	-5.545245
H	0.283184	-0.973052	-1.947653
H	-1.577959	-0.240454	-5.810614
H	-0.331856	0.456845	-7.569925
H	1.410034	0.890060	-7.340616
H	0.941448	-0.820072	-7.679319
H	6.532525	-1.196425	-0.846237
H	5.286205	-2.143874	0.026542
H	5.520931	0.926265	0.242761
H	6.406300	-0.202651	1.338251

H	5.687875	2.017018	2.206820
H	5.198012	3.396375	4.194024
H	1.965264	0.697889	5.303565
H	2.475127	-0.719284	3.282247
H	5.029388	3.825776	6.471027
H	3.856396	4.792011	5.504082
H	4.114553	5.456193	7.990632
H	3.394277	3.864039	8.423115
H	2.493102	7.237298	7.512094
H	2.409366	6.452318	5.907473
H	0.301490	7.820529	6.295693
H	-0.342345	5.109285	7.668368
H	-2.504725	6.492780	8.023683
H	-2.123088	7.423596	6.526828
H	-4.504227	6.331603	5.897691
H	-4.425409	5.270544	7.353191
H	-5.641036	3.949446	5.749567
H	-3.950145	3.318844	5.701529
H	-4.466681	4.972503	1.511089
H	-4.639855	3.243254	-0.317232
H	-5.013632	0.128424	2.713837
H	-4.893138	1.818874	4.486631
H	-5.695698	-1.031460	0.944722
H	-3.896187	-0.977203	0.779704
H	-5.997958	-1.287253	-1.489877
H	-4.957828	-2.591964	-0.829394
C	-6.784074	-4.382666	1.295553

C	-6.075837	-5.316768	0.509695
C	-4.662643	-5.403978	0.598634
C	-3.967774	-4.536238	1.477547
C	-4.669558	-3.573996	2.243615
C	-6.084624	-3.511583	2.156751
C	-3.977675	-6.413611	-0.294571
O	-4.660567	-7.126711	-1.114660
N	-2.612385	-6.522213	-0.194171
C	-1.824791	-7.435788	-1.035604
C	-0.679670	-6.721626	-1.752645
O	-1.264745	-5.861672	-2.804854
C	-0.362933	-5.092713	-3.561044
C	1.028375	-5.020086	-3.312778
C	1.861862	-4.252582	-4.161701
C	1.304446	-3.568370	-5.265417
C	-0.094560	-3.618246	-5.498054
C	-0.923896	-4.369288	-4.647423
O	2.056199	-2.824262	-6.199427
C	3.528199	-2.885513	-6.063080
C	4.136679	-2.131634	-7.229661
O	3.999580	-0.685970	-6.986472
C	4.477000	0.108421	-8.131535
C	4.541027	1.568330	-7.723150
O	3.171816	2.095416	-7.579703
C	3.190803	3.501603	-7.144090
C	1.758902	3.978871	-6.914163
O	1.028148	3.245326	-5.866765

C	1.710162	3.257783	-4.560341
C	0.629129	2.922243	-3.535661
O	1.340391	2.500813	-2.313798
C	0.564613	1.898466	-1.304126
C	-0.850844	1.810884	-1.336032
C	-1.535491	1.164081	-0.288160
C	-0.817843	0.608746	0.796573
C	0.597373	0.704321	0.831808
C	1.283180	1.350456	-0.217165
O	-1.589291	-0.015715	1.796832
C	-0.843007	-0.729842	2.853119
C	-1.858292	-1.424828	3.760198
N	-2.624355	-2.456281	3.044819
C	-3.990302	-2.548673	3.119430
O	-4.687481	-1.766368	3.866025
H	-7.875365	-4.326823	1.229353
H	-6.595470	-5.990364	-0.179092
H	-2.879150	-4.609427	1.568671
H	-6.612045	-2.768873	2.763630
H	-1.381920	-8.234985	-0.409563
H	-2.510463	-7.906089	-1.759140
H	-0.100331	-6.103771	-1.039888
H	-0.006236	-7.467930	-2.218744
H	1.483194	-5.551444	-2.473476
H	2.933256	-4.211842	-3.949487
H	-0.515472	-3.082479	-6.354290
H	-2.002934	-4.420264	-4.823270

H	3.842265	-2.436106	-5.101715
H	3.855384	-3.942920	-6.093263
H	5.213341	-2.392466	-7.307943
H	3.635085	-2.416680	-8.177457
H	5.495927	-0.226563	-8.419600
H	3.800567	-0.031311	-9.000131
H	5.081233	2.141931	-8.506293
H	5.089419	1.674540	-6.764082
H	3.653020	4.137969	-7.930039
H	3.802626	3.599345	-6.223332
H	1.793944	5.060607	-6.661438
H	1.153057	3.843318	-7.826019
H	2.509489	2.492184	-4.528769
H	2.157020	4.253218	-4.362150
H	-0.014587	3.795998	-3.319972
H	-0.002876	2.099721	-3.914878
H	-1.427345	2.226549	-2.166424
H	-2.624676	1.076673	-0.318321
H	1.175024	0.276636	1.652325
H	2.375284	1.418452	-0.203692
H	-0.252961	-0.009402	3.452050
H	-0.155244	-1.463740	2.388011
H	-2.574499	-0.699901	4.179058
H	-1.297443	-1.875997	4.602523
H	-2.078012	-5.946974	0.469593
H	-2.078949	-3.095076	2.451438
I	0.151136	-4.650039	1.646129

Table S10: Cartesian Coordenates of **1**.BF_4^-

**1**.BF_4^-	x	y	z
N	0.764264	0.451281	-5.405834
C	1.893387	0.386847	-4.642743
C	1.804074	0.060049	-3.273303
C	0.532512	-0.219044	-2.712389
C	-0.622768	-0.191627	-3.533003
C	-0.465915	0.170583	-4.889258
C	0.890337	0.743581	-6.862187
C	3.057342	0.063061	-2.515347
N	4.128980	0.837538	-2.976365
N	5.172870	0.710119	-2.133213
N	4.760254	-0.174075	-1.114071
C	3.458758	-0.590211	-1.326776
I	2.473129	-2.003215	-0.151399
C	5.725873	-0.489329	-0.046044
C	5.550831	0.382019	1.199429
O	4.416133	-0.183813	1.946981
C	4.087409	0.392323	3.188587
C	4.635671	1.598052	3.680671
C	4.225109	2.094987	4.943032
C	3.270680	1.381087	5.706836
C	2.723031	0.170214	5.204817
C	3.128334	-0.322748	3.953194
O	2.806745	1.775603	6.974625
C	3.331225	3.045481	7.518448
C	2.687412	3.256276	8.885944

O	1.239860	3.508724	8.824789
C	0.904374	4.882964	8.426655
C	-0.579620	4.990222	8.185801
C	-1.450261	3.949243	8.236621
C	-2.932497	4.095542	8.030408
O	-3.344836	3.161183	6.953231
C	-4.812552	3.075369	6.863561
C	-5.175892	2.102061	5.760462
O	-4.932766	2.769517	4.460248
C	-5.100789	1.977306	3.310689
C	-4.798900	2.599354	2.070289
C	-4.889642	1.865480	0.872274
C	-5.281343	0.503173	0.906302
C	-5.619669	-0.106696	2.134966
C	-5.532610	0.630858	3.336789
O	-5.307023	-0.180702	-0.330798
C	-5.254972	-1.651457	-0.223520
C	-4.902350	-2.223353	-1.596915
N	-3.824728	-1.502234	-2.301682
N	-4.130973	-1.024508	-3.592175
N	-3.002908	-0.474704	-4.075930
C	-1.978226	-0.583269	-3.126479
C	-2.509452	-1.230527	-1.982313
I	-1.633430	-1.721931	-0.153009
H	2.840670	0.554122	-5.155646
H	0.448702	-0.461668	-1.652855
H	-1.314069	0.214912	-5.571270

H	0.939954	-0.216000	-7.401458
H	0.008888	1.314933	-7.188501
H	1.809418	1.324812	-7.027563
H	6.725073	-0.314875	-0.474939
H	5.631964	-1.554802	0.220125
H	5.358083	1.435067	0.919482
H	6.469265	0.330300	1.815237
H	5.375974	2.160966	3.105196
H	4.662598	3.027868	5.308630
H	1.987790	-0.373334	5.806149
H	2.712032	-1.255932	3.562361
H	4.431404	2.976408	7.638248
H	3.100469	3.874320	6.821587
H	3.207889	4.092420	9.398657
H	2.796495	2.342051	9.493847
H	1.220648	5.593647	9.223367
H	1.449291	5.172938	7.501308
H	-0.940233	6.003339	7.953556
H	-1.082207	2.942002	8.466253
H	-3.483896	3.826748	8.959813
H	-3.204300	5.136181	7.754319
H	-5.250265	4.075925	6.662144
H	-5.226756	2.699014	7.823970
H	-6.247131	1.829811	5.837795
H	-4.562188	1.184164	5.840326
H	-4.473201	3.644317	2.060629
H	-4.645672	2.330800	-0.088131

H	-5.954375	-1.145904	2.178503
H	-5.799314	0.140113	4.274923
H	-6.237096	-2.060495	0.077914
H	-4.506771	-1.928666	0.536506
H	-5.764995	-2.170493	-2.278672
H	-4.623731	-3.283659	-1.462703
C	-6.343816	-3.598850	3.905409
C	-5.864764	-4.237870	2.740530
C	-4.529948	-4.027965	2.309302
C	-3.696389	-3.127962	3.017822
C	-4.189404	-2.459666	4.165340
C	-5.504803	-2.720966	4.624362
C	-4.071374	-4.780642	1.084680
O	-4.899191	-5.075245	0.147273
N	-2.754678	-5.165671	1.063068
C	-2.204421	-6.065416	0.035640
C	-1.066399	-5.469751	-0.796969
O	-1.647513	-4.755781	-1.959402
C	-0.722252	-4.215328	-2.874153
C	0.678031	-4.218798	-2.676943
C	1.536093	-3.654751	-3.650603
C	0.993802	-3.087809	-4.825536
C	-0.411215	-3.096089	-5.030124
C	-1.264448	-3.650206	-4.058973
O	1.768092	-2.485107	-5.842001
C	3.234849	-2.644307	-5.718583
C	3.899784	-1.966162	-6.902142

O	4.021076	-0.527726	-6.610757
C	4.662716	0.210865	-7.710444
C	4.938674	1.627849	-7.239554
O	3.655706	2.332371	-7.067562
C	3.856112	3.709199	-6.587057
C	2.496599	4.374322	-6.380418
O	1.651580	3.748216	-5.349330
C	2.296999	3.709670	-4.024568
C	1.180362	3.426145	-3.021844
O	1.860965	2.923618	-1.813125
C	1.054012	2.461586	-0.758354
C	-0.364668	2.474890	-0.777898
C	-1.078548	2.010551	0.344903
C	-0.387650	1.534702	1.483823
C	1.029602	1.504116	1.497170
C	1.744843	1.968878	0.373633
O	-1.195229	1.114249	2.556009
C	-0.513017	0.689314	3.801920
C	-1.628644	0.326248	4.775059
N	-2.467848	-0.728541	4.183857
C	-3.378613	-1.438073	4.919133
O	-3.562862	-1.225096	6.172194
H	-7.367502	-3.781584	4.247684
H	-6.506946	-4.912990	2.166001
H	-2.676444	-2.934653	2.671649
H	-5.860199	-2.213873	5.526999
H	-1.810737	-6.964446	0.546098

H	-3.031355	-6.376896	-0.623757
H	-0.454575	-4.775497	-0.191643
H	-0.418980	-6.286040	-1.172542
H	1.122333	-4.646444	-1.775824
H	2.612879	-3.665658	-3.465995
H	-0.822590	-2.663467	-5.947334
H	-2.348899	-3.651054	-4.206572
H	3.594511	-2.187811	-4.776832
H	3.478702	-3.724297	-5.711931
H	4.913482	-2.396034	-7.046037
H	3.312897	-2.131489	-7.828976
H	5.626463	-0.272334	-7.978377
H	4.007238	0.211493	-8.606050
H	5.556034	2.151549	-7.999928
H	5.493949	1.614475	-6.278776
H	4.422857	4.295381	-7.342687
H	4.448882	3.696387	-5.649318
H	2.672993	5.441275	-6.123376
H	1.897412	4.323647	-7.305182
H	3.049039	2.899772	-3.975295
H	2.794172	4.677601	-3.808840
H	0.598323	4.335600	-2.780011
H	0.492797	2.661364	-3.427050
H	-0.917101	2.849037	-1.643799
H	-2.171892	2.013193	0.348756
H	1.582482	1.132929	2.363390
H	2.838928	1.959092	0.369773

H	0.094173	1.521060	4.203195
H	0.135757	-0.177768	3.586079
H	-2.236199	1.221187	5.009809
H	-1.191008	-0.035789	5.721642
H	-2.137333	-4.934531	1.853964
H	-2.456640	-0.804056	3.162081
F	1.213588	-3.338520	3.796406
F	-0.628843	-4.587201	2.992860
F	-0.352235	-2.322331	2.343068
F	1.123442	-3.990455	1.519030
B	0.346671	-3.555823	2.671706

Table S11: Cartesian Coordenates of **1**.HSO_4^-

**1**.HSO_4^-	x	y	z
N	0.726946	0.099492	-5.698472
C	1.862608	-0.121458	-4.973068
C	1.771847	-0.485413	-3.611570
C	0.493064	-0.625044	-3.023518
C	-0.673273	-0.407120	-3.798897
C	-0.519049	-0.043344	-5.152399
C	0.869750	0.460820	-7.138168
C	3.028166	-0.636536	-2.874765
N	4.182115	-0.001426	-3.349984
N	5.212852	-0.258408	-2.517412
N	4.702334	-1.079668	-1.490860
C	3.356831	-1.327543	-1.683651
I	2.184169	-2.554075	-0.462021

C	5.612557	-1.468361	-0.398888
C	5.635826	-0.432579	0.727162
O	4.394337	-0.620374	1.497698
C	4.158905	0.242582	2.588455
C	4.932424	1.387947	2.885089
C	4.591014	2.202234	3.994079
C	3.480359	1.864740	4.804243
C	2.716238	0.704064	4.507919
C	3.053674	-0.104953	3.408779
O	3.058639	2.603179	5.926287
C	3.824726	3.825425	6.250756
C	3.194388	4.456129	7.490212
O	1.855982	5.025548	7.270292
C	1.879108	6.307041	6.551058
C	0.471680	6.822448	6.388301
C	-0.643397	6.185585	6.829417
C	-2.041521	6.711296	6.643118
O	-2.785765	5.694205	5.859447
C	-4.243608	5.903103	5.894027
C	-4.876275	4.770955	5.103883
O	-4.733541	5.082142	3.663714
C	-4.885933	4.021075	2.750426
C	-4.680584	4.354117	1.384731
C	-4.754349	3.355467	0.395994
C	-5.027398	2.013305	0.764984
C	-5.263187	1.679618	2.116484
C	-5.199098	2.687555	3.109201

O	-5.035316	1.076901	-0.296965
C	-4.967400	-0.349386	0.080682
C	-5.005908	-1.159712	-1.215291
N	-3.926587	-0.805342	-2.153316
N	-4.224192	0.035569	-3.243505
N	-3.063657	0.221413	-3.909277
C	-2.035906	-0.479562	-3.265071
C	-2.587783	-1.137383	-2.140532
I	-1.726821	-2.414858	-0.731427
H	2.811879	-0.023971	-5.507525
H	0.404658	-0.860935	-1.963597
H	-1.372532	0.144367	-5.803374
H	1.013281	-0.464400	-7.719380
H	-0.043615	0.974853	-7.470117
H	1.743666	1.122146	-7.248777
H	6.619145	-1.552578	-0.837995
H	5.305695	-2.453005	-0.011866
H	5.691713	0.590276	0.309514
H	6.513164	-0.610425	1.378130
H	5.795871	1.665757	2.274038
H	5.201379	3.084078	4.206507
H	1.866971	0.446451	5.148638
H	2.471263	-1.004628	3.178639
H	4.876666	3.556712	6.476330
H	3.813618	4.515902	5.385190
H	3.884467	5.237091	7.874522
H	3.055627	3.687797	8.269874

H	2.501237	7.042193	7.110638
H	2.342660	6.188461	5.547082
H	0.388399	7.780962	5.854832
H	-0.548878	5.225577	7.351006
H	-2.546478	6.845932	7.625743
H	-2.047559	7.684600	6.109322
H	-4.514983	6.888897	5.462071
H	-4.606377	5.865556	6.943778
H	-5.951266	4.667455	5.347532
H	-4.354051	3.827268	5.349398
H	-4.447069	5.389312	1.115950
H	-4.578591	3.598827	-0.656809
H	-5.460052	0.649324	2.432451
H	-5.385338	2.406481	4.149318
H	-5.844116	-0.635291	0.688778
H	-4.045772	-0.528398	0.664396
H	-5.949728	-0.979670	-1.753151
H	-4.932593	-2.232227	-0.969433
C	-6.605413	-4.397653	0.725306
C	-5.675418	-5.342799	0.243978
C	-4.320448	-5.294199	0.664069
C	-3.908958	-4.289278	1.573811
C	-4.836761	-3.328872	2.051214
C	-6.188063	-3.393373	1.623454
C	-3.394335	-6.343723	0.095265
O	-3.877220	-7.386149	-0.479163
N	-2.039810	-6.136843	0.202906

C	-1.068638	-7.087171	-0.361233
C	-0.009090	-6.398007	-1.218829
O	-0.701703	-5.747932	-2.353481
C	0.100227	-5.005462	-3.237705
C	1.513778	-4.984523	-3.198171
C	2.239465	-4.227906	-4.148298
C	1.550728	-3.496998	-5.142934
C	0.132469	-3.508200	-5.175948
C	-0.588720	-4.253416	-4.226205
O	2.186918	-2.732943	-6.144686
C	3.658783	-2.860318	-6.227641
C	4.138789	-2.045361	-7.412071
O	4.151457	-0.625370	-7.026448
C	4.625748	0.240266	-8.118939
C	4.872351	1.627619	-7.556442
O	3.576641	2.252299	-7.239483
C	3.762104	3.551916	-6.574197
C	2.394943	4.163333	-6.283454
O	1.561483	3.380841	-5.357358
C	2.160466	3.228383	-4.018252
C	0.983855	2.924619	-3.095273
O	1.553206	2.354247	-1.860787
C	0.630933	1.860743	-0.914557
C	-0.775790	1.884493	-1.095052
C	-1.617230	1.345838	-0.102478
C	-1.068487	0.785348	1.074281
C	0.337440	0.774532	1.261198

C	1.181769	1.309370	0.264987
O	-1.993480	0.247493	1.989720
C	-1.458210	-0.274098	3.264996
C	-2.627057	-0.888736	4.041581
N	-3.163232	-2.083498	3.370821
C	-4.460826	-2.168071	2.942180
O	-5.338979	-1.273290	3.232996
H	-7.649118	-4.440073	0.397612
H	-5.976798	-6.129832	-0.454302
H	-2.873498	-4.267695	1.925409
H	-6.889539	-2.643702	2.002433
H	-0.542494	-7.627768	0.450167
H	-1.632860	-7.825300	-0.953963
H	0.538414	-5.632297	-0.637707
H	0.704534	-7.158036	-1.592200
H	2.066545	-5.542009	-2.438760
H	3.330833	-4.222642	-4.092389
H	-0.391038	-2.938530	-5.949736
H	-1.682944	-4.267785	-4.243342
H	4.129806	-2.491912	-5.296219
H	3.923474	-3.925716	-6.372127
H	5.169113	-2.364412	-7.676586
H	3.482362	-2.207985	-8.291591
H	5.578848	-0.157955	-8.526519
H	3.876660	0.270418	-8.937166
H	5.409188	2.242800	-8.309462
H	5.496865	1.561516	-6.641344

H	4.323688	4.243615	-7.239547
H	4.353897	3.414842	-5.645235
H	2.550884	5.185901	-5.877301
H	1.799379	4.232801	-7.209520
H	2.885041	2.391408	-4.001907
H	2.680696	4.160080	-3.717299
H	0.405828	3.838365	-2.859702
H	0.312406	2.197110	-3.584363
H	-1.228258	2.310492	-1.993783
H	-2.699809	1.348698	-0.247373
H	0.787704	0.353144	2.161493
H	2.268171	1.296373	0.397162
H	-1.018669	0.556773	3.850938
H	-0.669386	-1.025665	3.067938
H	-3.444900	-0.159099	4.149253
H	-2.258751	-1.151659	5.051573
H	-1.644595	-5.276866	0.604407
H	-2.493501	-2.864460	3.246593
S	0.237423	-3.888471	2.564743
O	-1.175135	-4.053277	3.395298
O	1.155696	-2.611778	3.022961
O	1.200943	-5.421270	2.884586
O	0.027107	-4.053515	0.923854
H	1.410017	-5.456331	3.867869

Table S12: Cartesian Coordenates of **1**.MS^{-}

$\mathbf{1}$.MS$^-$	x	y	z
N	1.038259	-0.058159	-5.627726
C	2.121976	-0.139844	-4.801798
C	1.949409	-0.442015	-3.433875
C	0.641971	-0.678821	-2.941078
C	-0.467227	-0.616427	-3.821199
C	-0.226881	-0.288648	-5.174116
C	1.254172	0.208489	-7.078434
C	3.158340	-0.455626	-2.606040
N	4.278887	0.265597	-3.040310
N	5.278738	0.125463	-2.147447
N	4.784005	-0.710380	-1.123069
C	3.476202	-1.082500	-1.376600
I	2.387079	-2.429713	-0.174364
C	5.689100	-1.031616	-0.006138
C	5.608089	-0.016138	1.134132
O	4.389074	-0.331415	1.899908
C	4.116393	0.452231	3.038482
C	4.801455	1.640885	3.377835
C	4.425341	2.364053	4.537331
C	3.367757	1.895123	5.353526
C	2.689629	0.694184	5.012435
C	3.064363	-0.022974	3.863694
O	2.919612	2.534674	6.522584
C	3.539832	3.834035	6.860384
C	2.832253	4.369932	8.102246
O	1.431267	4.751838	7.867450

C	1.285589	6.053376	7.199466
C	-0.162454	6.279968	6.846623
C	-1.157331	5.378413	7.048545
C	-2.600170	5.629681	6.704795
O	-3.035262	4.553480	5.779797
C	-4.502668	4.484866	5.671392
C	-4.856013	3.337789	4.744293
O	-4.653631	3.789632	3.348399
C	-4.853299	2.820488	2.346258
C	-4.676390	3.259836	1.007227
C	-4.817282	2.351828	-0.059984
C	-5.137958	0.995875	0.204175
C	-5.343149	0.564670	1.534000
C	-5.201674	1.474281	2.606774
O	-5.225196	0.137061	-0.913689
C	-5.246620	-1.304082	-0.596404
C	-4.967582	-2.081169	-1.883099
N	-3.804039	-1.575151	-2.635993
N	-4.039412	-1.129345	-3.954174
N	-2.849386	-0.730666	-4.440178
C	-1.859475	-0.904342	-3.462265
C	-2.471019	-1.441367	-2.302456
I	-1.671183	-1.974132	-0.428684
H	3.100280	-0.003751	-5.264913
H	0.493569	-0.909337	-1.885512
H	-1.037507	-0.222684	-5.898977
H	1.329506	-0.759677	-7.598927

H	0.398214	0.780417	-7.465856
H	2.185424	0.781968	-7.198357
H	6.710490	-1.028379	-0.418883
H	5.457153	-2.043484	0.363016
H	5.570337	1.015426	0.736104
H	6.495968	-0.122544	1.786769
H	5.621389	2.020913	2.762177
H	4.967529	3.280631	4.785112
H	1.877830	0.336512	5.653381
H	2.551248	-0.948749	3.588453
H	4.615899	3.687215	7.084184
H	3.446055	4.527219	6.002067
H	3.411170	5.232381	8.495001
H	2.792901	3.585546	8.877417
H	1.655030	6.863054	7.868787
H	1.899653	6.095249	6.272968
H	-0.381699	7.255564	6.387868
H	-0.925181	4.406406	7.500247
H	-3.231987	5.587660	7.620452
H	-2.742235	6.622207	6.228221
H	-4.916629	5.440750	5.287091
H	-4.942718	4.289270	6.673164
H	-5.915463	3.045181	4.882545
H	-4.209316	2.465282	4.959966
H	-4.413109	4.305106	0.816193
H	-4.666153	2.678459	-1.093849
H	-5.614363	-0.468736	1.757489

H	-5.344092	1.101332	3.624246
H	-6.239504	-1.603727	-0.211401
H	-4.486784	-1.519037	0.173218
H	-5.815619	-2.007019	-2.581593
H	-4.819759	-3.144821	-1.622130
C	-6.855263	-3.423739	2.748152
C	-6.274499	-4.199874	1.722363
C	-4.875283	-4.148228	1.494533
C	-4.067901	-3.286032	2.277192
C	-4.653640	-2.472785	3.280462
C	-6.048632	-2.563641	3.522861
C	-4.330035	-4.993756	0.368415
O	-5.073976	-5.272720	-0.645159
N	-3.048878	-5.456135	0.500502
C	-2.430324	-6.361609	-0.481717
C	-1.207279	-5.778902	-1.196777
O	-1.670950	-4.994882	-2.368518
C	-0.661085	-4.500446	-3.215207
C	0.717104	-4.521046	-2.897230
C	1.668535	-4.021559	-3.817770
C	1.241231	-3.498172	-5.058846
C	-0.141424	-3.484909	-5.382024
C	-1.086996	-3.976560	-4.464472
O	2.110401	-2.960784	-6.033787
C	3.559278	-3.117438	-5.775656
C	4.323884	-2.467974	-6.914161
O	4.392655	-1.018743	-6.661624

C	5.086206	-0.296716	-7.740632
C	5.306054	1.138004	-7.294201
O	4.003094	1.823169	-7.226475
C	4.144759	3.210791	-6.756539
C	2.759461	3.841902	-6.627453
O	1.882425	3.209416	-5.627345
C	2.462452	3.205660	-4.272579
C	1.297766	2.954905	-3.316460
O	1.918340	2.476800	-2.067336
C	1.060332	2.078390	-1.026130
C	-0.355696	2.153239	-1.085208
C	-1.120503	1.749619	0.028160
C	-0.484260	1.270141	1.197566
C	0.930402	1.185486	1.250618
C	1.696963	1.590125	0.138375
O	-1.336351	0.896741	2.251223
C	-0.700030	0.347551	3.468037
C	-1.821745	-0.122051	4.388917
N	-2.576859	-1.229228	3.776195
C	-3.894297	-1.437136	4.079436
O	-4.496798	-0.744842	4.983161
H	-7.932737	-3.479635	2.934515
H	-6.888672	-4.850679	1.091961
H	-2.995847	-3.229217	2.072085
H	-6.480964	-1.932847	4.305793
H	-2.099596	-7.272638	0.051960
H	-3.195907	-6.650017	-1.220802

H	-0.612379	-5.138812	-0.517466
H	-0.564714	-6.605338	-1.560511
H	1.071652	-4.917566	-1.943649
H	2.725493	-4.048406	-3.541615
H	-0.462976	-3.089838	-6.350471
H	-2.153967	-3.969346	-4.707543
H	3.836267	-2.635708	-4.818450
H	3.800573	-4.196735	-5.719669
H	5.353284	-2.883552	-6.946621
H	3.829931	-2.674790	-7.885899
H	6.073769	-0.766297	-7.936701
H	4.485998	-0.334173	-8.673488
H	5.963346	1.652367	-8.027194
H	5.797025	1.161014	-6.299078
H	4.734554	3.801855	-7.490645
H	4.688728	3.225729	-5.789531
H	2.894056	4.917523	-6.381243
H	2.207908	3.758656	-7.579194
H	3.209474	2.396040	-4.163707
H	2.952806	4.177606	-4.058868
H	0.715011	3.876212	-3.125382
H	0.620874	2.186219	-3.732643
H	-0.867439	2.522682	-1.977984
H	-2.212792	1.797785	-0.000981
H	1.443715	0.821812	2.142741
H	2.789318	1.534639	0.168951
H	-0.105924	1.135187	3.968723

H	-0.038308	-0.493770	3.188534
H	-2.521303	0.703429	4.606920
H	-1.369528	-0.433727	5.352229
H	-2.477477	-5.262198	1.352290
H	-2.056933	-1.805541	3.085756
S	-0.058638	-3.998725	2.369442
O	-1.215074	-5.172403	2.578141
O	-0.750761	-2.492541	2.061746
O	1.070954	-4.388525	1.208236
C	0.843946	-3.803786	4.017850
H	0.090112	-3.488289	4.752891
H	1.273967	-4.786986	4.257158
H	1.618513	-3.041717	3.857178

Table S13: Cartesian Coordenates of **1**.TS^{-}

**1**.TS^{-}	x	y	z
N	1.123936	0.335161	-5.995360
C	2.166381	0.202007	-5.125384
C	1.922990	-0.095994	-3.767791
C	0.586503	-0.270473	-3.328395
C	-0.479945	-0.162089	-4.256811
C	-0.167343	0.159707	-5.595765
C	1.406446	0.587119	-7.436919
C	3.096332	-0.182305	-2.895001
N	4.263348	0.506181	-3.252315
N	5.216189	0.288533	-2.323579
N	4.642459	-0.565795	-1.356459

C	3.334217	-0.871231	-1.682413
I	2.134667	-2.224807	-0.601487
C	5.483761	-0.991590	-0.224906
C	5.381029	-0.066805	0.988301
O	4.112166	-0.383924	1.666097
C	3.833135	0.295928	2.868821
C	4.603094	1.364331	3.383488
C	4.220304	1.987820	4.597809
C	3.071316	1.537699	5.291193
C	2.306381	0.461950	4.770186
C	2.684500	-0.155974	3.567595
O	2.605005	2.078316	6.502287
C	3.247672	3.318802	6.983403
C	2.453484	3.798288	8.196169
O	1.077345	4.209952	7.880080
C	0.994091	5.526578	7.233195
C	-0.428221	5.793814	6.811574
C	-1.451132	4.907258	6.920064
C	-2.868119	5.220709	6.524238
O	-3.308576	4.208840	5.531463
C	-4.767377	4.264933	5.328394
C	-5.158845	3.216310	4.306059
O	-4.800178	3.726932	2.961204
C	-5.017488	2.852249	1.880499
C	-4.682901	3.352806	0.594206
C	-4.825131	2.533482	-0.542406
C	-5.305700	1.207200	-0.400450

C	-5.671670	0.718958	0.873768
C	-5.529494	1.540017	2.015198
O	-5.384982	0.433683	-1.579806
C	-5.475163	-1.024561	-1.370075
C	-5.086774	-1.716312	-2.677879
N	-3.882442	-1.148634	-3.312350
N	-4.030546	-0.666359	-4.630080
N	-2.816514	-0.236911	-5.017893
C	-1.895688	-0.425756	-3.977524
C	-2.580680	-1.000941	-2.877936
I	-1.905094	-1.554055	-0.959540
H	3.168192	0.291355	-5.544905
H	0.381490	-0.493438	-2.280874
H	-0.942932	0.258222	-6.354463
H	1.402435	-0.381724	-7.961789
H	0.619563	1.238992	-7.843957
H	2.389872	1.071518	-7.525032
H	6.523182	-0.988653	-0.590023
H	5.208125	-2.021026	0.055814
H	5.406666	0.994895	0.678232
H	6.229821	-0.267437	1.670047
H	5.493471	1.726132	2.861935
H	4.827713	2.811333	4.983374
H	1.420892	0.123302	5.314697
H	2.099486	-0.982617	3.157104
H	4.293074	3.109387	7.287935
H	3.258561	4.072926	6.172764

H	3.009959	4.630409	8.677214
H	2.346664	2.974530	8.922326
H	1.344820	6.314904	7.937015
H	1.656160	5.573076	6.340843
H	-0.605126	6.792846	6.386114
H	-1.266109	3.911946	7.341797
H	-3.540179	5.161985	7.410041
H	-2.953933	6.239091	6.090104
H	-5.076329	5.275984	4.988493
H	-5.286148	4.044033	6.286369
H	-6.252212	3.040392	4.347159
H	-4.635004	2.262231	4.508181
H	-4.294895	4.372149	0.500550
H	-4.554268	2.903684	-1.536367
H	-6.073516	-0.288865	0.997829
H	-5.801326	1.135083	2.992572
H	-6.508148	-1.319008	-1.107259
H	-4.800981	-1.315926	-0.548569
H	-5.881739	-1.612144	-3.432481
H	-4.938232	-2.790286	-2.467808
C	-7.109398	-2.790208	2.501223
C	-6.607320	-3.551284	1.423482
C	-5.218952	-3.541607	1.131984
C	-4.346080	-2.729882	1.896304
C	-4.853262	-1.932916	2.953845
C	-6.235979	-1.986818	3.264720
C	-4.750137	-4.379049	-0.033469

O	-5.530467	-4.587164	-1.035809
N	-3.494578	-4.919498	0.059209
C	-2.967907	-5.867841	-0.937458
C	-1.693674	-5.405023	-1.649959
O	-2.074327	-4.594490	-2.833616
C	-1.008710	-4.145672	-3.637377
C	0.355572	-4.273108	-3.286438
C	1.363881	-3.796657	-4.158113
C	1.008776	-3.194529	-5.386142
C	-0.360198	-3.076402	-5.744013
C	-1.362121	-3.542470	-4.873948
O	1.940733	-2.673717	-6.312291
C	3.366664	-2.942397	-6.020766
C	4.211871	-2.344833	-7.130886
O	4.407013	-0.911211	-6.856409
C	5.212988	-0.249756	-7.895315
C	5.522379	1.163981	-7.434732
O	4.276811	1.951411	-7.442357
C	4.502486	3.331900	-6.984472
C	3.165129	4.069354	-6.931819
O	2.195151	3.525095	-5.966515
C	2.709870	3.508023	-4.586132
C	1.495783	3.324444	-3.676881
O	2.048259	2.808995	-2.411081
C	1.146088	2.416804	-1.406996
C	-0.264203	2.538679	-1.502580
C	-1.070809	2.136856	-0.417755

C	-0.482420	1.613749	0.758177
C	0.927345	1.478384	0.844939
C	1.734786	1.881386	-0.237949
O	-1.370703	1.257439	1.788337
C	-0.763158	0.695458	3.014542
C	-1.900295	0.306364	3.949590
N	-2.716822	-0.767272	3.358441
C	-4.018905	-0.945316	3.733995
O	-4.545219	-0.254098	4.685593
H	-8.178195	-2.814521	2.737586
H	-7.274422	-4.160757	0.805573
H	-3.284079	-2.700936	1.637968
H	-6.607741	-1.371863	4.090251
H	-2.731712	-6.815004	-0.416130
H	-3.761101	-6.070227	-1.676259
H	-1.046603	-4.815281	-0.973258
H	-1.126820	-6.289836	-2.001801
H	0.656287	-4.732804	-2.342467
H	2.407948	-3.901028	-3.853497
H	-0.627246	-2.617099	-6.700840
H	-2.419064	-3.445489	-5.140782
H	3.652140	-2.495278	-5.049408
H	3.522900	-4.037612	-5.973310
H	5.200050	-2.851496	-7.147282
H	3.724206	-2.492885	-8.116389
H	6.168300	-0.798351	-8.039413
H	4.662449	-0.240149	-8.858978

H	6.259921	1.621213	-8.127844
H	5.954996	1.151057	-6.412786
H	5.169639	3.862872	-7.697798
H	5.000762	3.322215	-5.993223
H	3.371281	5.135784	-6.695494
H	2.654995	4.011674	-7.908180
H	3.407021	2.661969	-4.435101
H	3.240416	4.455783	-4.360167
H	0.958949	4.275793	-3.499933
H	0.792144	2.596923	-4.121263
H	-0.739150	2.945832	-2.399376
H	-2.159236	2.227387	-0.471309
H	1.406211	1.078481	1.740841
H	2.823648	1.790604	-0.181982
H	-0.122480	1.455842	3.498245
H	-0.150691	-0.184566	2.745371
H	-2.550070	1.172657	4.161087
H	-1.459836	-0.013620	4.915056
H	-2.906495	-4.779760	0.908613
H	-2.255215	-1.344380	2.628640
S	-0.410314	-3.646687	1.843539
O	-1.616998	-4.739451	2.156224
O	-1.056086	-2.122187	1.540383
O	0.604758	-4.132905	0.618001
C	0.651415	-3.437765	3.375198
C	0.061071	-2.893591	4.530758
C	1.998567	-3.824145	3.301370

H	-0.996326	-2.612909	4.560285
H	2.412198	-4.248363	2.381703
C	0.884394	-2.713987	5.661822
C	2.801106	-3.632012	4.449044
H	0.452514	-2.286850	6.573157
H	3.858249	-3.915734	4.414801
C	2.261994	-3.069757	5.635989
C	3.141656	-2.802039	6.839208
H	2.620786	-3.037961	7.784188
H	4.077755	-3.384383	6.798418
H	3.416080	-1.729390	6.879475

Table S14: Cartesian Coordenates of **2**.TS^{-}

**2**.TS^{-}	x	y	z
N	1.123851	0.346954	-5.979132
C	2.165629	0.210327	-5.108690
C	1.921231	-0.095146	-3.752749
C	0.584070	-0.272872	-3.316059
C	-0.481621	-0.162103	-4.245404
C	-0.167741	0.167672	-5.582080
C	1.407021	0.606363	-7.419439
C	3.094172	-0.188097	-2.879434
N	4.263945	0.497624	-3.233974
N	5.216880	0.271374	-2.307250
N	4.640340	-0.585230	-1.343398
C	3.330425	-0.883515	-1.669996
C	5.481312	-1.021289	-0.215469

C	5.389365	-0.098729	1.000300
O	4.115814	-0.402114	1.675730
C	3.841107	0.281448	2.877332
C	4.624227	1.338075	3.396450
C	4.244061	1.966795	4.608901
C	3.084749	1.533549	5.295768
C	2.306914	0.469173	4.770555
C	2.682298	-0.153699	3.569738
O	2.619683	2.081212	6.504156
C	3.269310	3.319020	6.982688
C	2.464852	3.817469	8.181206
O	1.092192	4.227666	7.848285
C	1.017985	5.523010	7.158738
C	-0.404947	5.791827	6.740135
C	-1.434925	4.917560	6.877986
C	-2.851028	5.233356	6.480899
O	-3.300732	4.208613	5.505054
C	-4.759906	4.271245	5.305151
C	-5.158854	3.220986	4.287248
O	-4.798704	3.725485	2.940319
C	-5.019705	2.848270	1.862505
C	-4.684942	3.344250	0.574485
C	-4.829947	2.521911	-0.559609
C	-5.313732	1.197316	-0.413121
C	-5.680488	0.713890	0.862719
C	-5.535310	1.537893	2.001599
O	-5.396077	0.420128	-1.589924

C	-5.483155	-1.037879	-1.375140
C	-5.087252	-1.733456	-2.679088
N	-3.883752	-1.161675	-3.311059
N	-4.030037	-0.678696	-4.628881
N	-2.816496	-0.243993	-5.012796
C	-1.897566	-0.430302	-3.969994
C	-2.583755	-1.008771	-2.872996
H	3.167536	0.302835	-5.527252
H	0.378050	-0.502728	-2.270212
H	-0.942295	0.267146	-6.341713
H	1.403393	-0.360074	-7.948919
H	0.619928	1.259986	-7.823491
H	2.390404	1.091442	-7.505333
H	6.519869	-1.026174	-0.583210
H	5.197547	-2.049103	0.063229
H	5.428263	0.963555	0.693234
H	6.235162	-0.311737	1.682195
H	5.522614	1.686958	2.879826
H	4.861676	2.780796	4.998548
H	1.413542	0.143331	5.309994
H	2.087183	-0.971007	3.155090
H	4.308655	3.102030	7.302571
H	3.298655	4.066017	6.165815
H	3.019510	4.655290	8.654896
H	2.348917	3.004850	8.918493
H	1.383898	6.330494	7.832672
H	1.671944	5.531644	6.259041

H	-0.575897	6.780580	6.288844
H	-1.255927	3.931740	7.324017
H	-3.521231	5.193349	7.369176
H	-2.931525	6.245025	6.030147
H	-5.064390	5.282536	4.961691
H	-5.277646	4.056941	6.265238
H	-6.253308	3.051667	4.330181
H	-4.640720	2.264233	4.492015
H	-4.294318	4.362298	0.477614
H	-4.558233	2.888184	-1.554771
H	-6.084452	-0.292745	0.990054
H	-5.807924	1.136393	2.980209
H	-6.516387	-1.334185	-1.115014
H	-4.810897	-1.324394	-0.550175
H	-5.880180	-1.636563	-3.436874
H	-4.933253	-2.805697	-2.463987
C	-7.116178	-2.798521	2.496190
C	-6.611198	-3.561461	1.421125
C	-5.222398	-3.550082	1.131563
C	-4.351994	-2.735345	1.895546
C	-4.862090	-1.936546	2.950386
C	-6.245270	-1.991720	3.258991
C	-4.750929	-4.388118	-0.032424
O	-5.530308	-4.598729	-1.035004
N	-3.493998	-4.925594	0.060753
C	-2.965065	-5.872521	-0.936364
C	-1.692918	-5.406398	-1.650860

O	-2.076614	-4.597212	-2.834897
C	-1.012412	-4.145201	-3.638907
C	0.352326	-4.269177	-3.288374
C	1.359127	-3.788110	-4.159198
C	1.002112	-3.184732	-5.386067
C	-0.367201	-3.070405	-5.743812
C	-1.367700	-3.540687	-4.874303
O	1.932796	-2.660466	-6.311713
C	3.359339	-2.928830	-6.021516
C	4.204235	-2.328000	-7.130341
O	4.402832	-0.895689	-6.850075
C	5.209901	-0.232429	-7.887359
C	5.521875	1.179724	-7.423314
O	4.277490	1.969235	-7.429657
C	4.505196	3.349308	-6.971087
C	3.168390	4.087811	-6.916503
O	2.197879	3.540705	-5.953044
C	2.712559	3.516778	-4.572588
C	1.497714	3.329984	-3.664767
O	2.049018	2.811120	-2.399717
C	1.145307	2.417749	-1.397488
C	-0.264827	2.540045	-1.495233
C	-1.073206	2.135425	-0.412834
C	-0.486568	1.610579	0.763134
C	0.922958	1.474559	0.851988
C	1.732151	1.879573	-0.228814
O	-1.376691	1.253055	1.791241

C	-0.771407	0.691282	3.018911
C	-1.910908	0.303174	3.951761
N	-2.726518	-0.770670	3.359577
C	-4.030162	-0.946738	3.730545
O	-4.559661	-0.252552	4.678246
H	-8.185285	-2.824155	2.731055
H	-7.276378	-4.173308	0.803496
H	-3.289556	-2.705659	1.639155
H	-6.619344	-1.375231	4.082344
H	-2.725116	-6.818725	-0.414908
H	-3.758615	-6.077743	-1.674078
H	-1.046328	-4.814824	-0.975066
H	-1.124423	-6.290230	-2.003025
H	0.654534	-4.728173	-2.344558
H	2.403544	-3.889986	-3.854877
H	-0.635783	-2.608316	-6.698898
H	-2.424948	-3.446309	-5.140900
H	3.644875	-2.483978	-5.049029
H	3.516188	-4.024180	-5.976885
H	5.191527	-2.836470	-7.150207
H	3.714955	-2.470876	-8.115921
H	6.164269	-0.782423	-8.033167
H	4.659146	-0.219218	-8.850972
H	6.260418	1.637224	-8.115322
H	5.954205	1.163835	-6.401193
H	5.172236	3.880223	-7.684709
H	5.004429	3.338457	-5.980221

H	3.375216	5.153391	-6.676592
H	2.658166	4.033643	-7.893094
H	3.408972	2.669260	-4.425156
H	3.243915	4.463083	-4.342002
H	0.960306	4.280770	-3.485687
H	0.794714	2.603376	-4.111953
H	-0.738297	2.948634	-2.392149
H	-2.161604	2.226226	-0.466884
H	1.400281	1.073894	1.748373
H	2.820888	1.788122	-0.171594
H	-0.131614	1.452013	3.503576
H	-0.158670	-0.189234	2.751401
H	-2.560619	1.170238	4.160484
H	-1.472810	-0.016217	4.918609
H	-2.907946	-4.786227	0.911529
H	-2.262821	-1.349537	2.632452
S	-0.416715	-3.651484	1.848973
O	-1.622446	-4.743466	2.167527
O	-1.063191	-2.127383	1.545549
O	0.592668	-4.140296	0.619597
C	0.653827	-3.438708	3.374264
C	0.070514	-2.892053	4.532229
C	2.001173	-3.822897	3.292253
H	-0.987041	-2.612658	4.567901
H	2.409423	-4.248921	2.371019
C	0.900872	-2.709437	5.657758
C	2.810777	-3.627733	4.434417

H	0.474385	-2.281033	6.571045
H	3.868053	-3.910218	4.393836
C	2.278570	-3.064232	5.623880
C	3.165772	-2.794487	6.821158
H	2.650534	-3.027708	7.769903
H	4.101195	-3.377650	6.775945
H	3.441376	-1.721982	6.857181
Br	-1.856100	-1.481538	-1.137885
Br	2.090776	-2.006797	-0.687597

Table S15: Cartesian Coordenates of **3**.TS^{-}

**3**.TS^{-}	x	y	z
N	1.136617	0.207823	-5.956661
C	2.172562	0.105068	-5.071899
C	1.918079	-0.197789	-3.718925
C	0.582328	-0.398070	-3.289461
C	-0.471071	-0.310271	-4.230007
C	-0.155148	0.000908	-5.569941
C	1.435385	0.456070	-7.396225
C	3.070667	-0.296119	-2.826208
N	4.279497	0.330715	-3.146025
N	5.197632	0.062099	-2.190903
N	4.559905	-0.761344	-1.238727
C	3.254402	-0.982323	-1.611600
C	5.333489	-1.244811	-0.081514
C	5.270254	-0.297888	1.116094
O	3.949213	-0.485695	1.739587

C	3.695063	0.225953	2.931327
C	4.528741	1.242481	3.450958
C	4.166065	1.904910	4.650603
C	2.974888	1.543907	5.325016
C	2.146592	0.518341	4.798828
C	2.504647	-0.136670	3.609813
O	2.526538	2.129139	6.522105
C	3.254374	3.320728	7.006768
C	2.500737	3.849900	8.224722
O	1.152243	4.350876	7.919167
C	1.147436	5.673226	7.277959
C	-0.255535	6.019255	6.847244
C	-1.324075	5.187729	6.950581
C	-2.721494	5.562437	6.537686
O	-3.189784	4.555565	5.552220
C	-4.654750	4.587682	5.393458
C	-5.050303	3.500337	4.412780
O	-4.789670	3.993578	3.039989
C	-4.972897	3.063678	1.998508
C	-4.739786	3.543463	0.682146
C	-4.848347	2.671063	-0.418009
C	-5.188863	1.310632	-0.208867
C	-5.455300	0.840485	1.096046
C	-5.351625	1.715777	2.200987
O	-5.231808	0.485082	-1.356041
C	-5.258888	-0.964887	-1.083191
C	-5.040380	-1.692856	-2.407769

N	-3.861326	-1.208705	-3.149454
N	-4.055360	-0.744961	-4.465927
N	-2.844540	-0.365568	-4.925023
C	-1.878921	-0.567428	-3.932345
C	-2.536121	-1.098480	-2.805468
H	3.178596	0.211302	-5.482589
H	0.371361	-0.627130	-2.244552
H	-0.925697	0.077759	-6.336684
H	1.518195	-0.519695	-7.901442
H	0.613108	1.040978	-7.833444
H	2.383542	1.009155	-7.473234
H	6.378154	-1.333852	-0.419295
H	4.964575	-2.244241	0.200229
H	5.409918	0.751691	0.795403
H	6.067687	-0.568063	1.834718
H	5.452822	1.535658	2.945110
H	4.821921	2.688469	5.040007
H	1.230148	0.244977	5.329414
H	1.874933	-0.927696	3.195655
H	4.283590	3.038623	7.308111
H	3.315286	4.074538	6.197809
H	3.114375	4.643144	8.702079
H	2.345277	3.034288	8.951426
H	1.536722	6.437687	7.987969
H	1.817050	5.686625	6.389947
H	-0.374487	7.024940	6.417289
H	-1.192554	4.185215	7.375567

H	-3.403983	5.541482	7.416917
H	-2.759630	6.578134	6.091250
H	-4.992499	5.582372	5.034006
H	-5.139768	4.385099	6.372710
H	-6.127686	3.263378	4.513908
H	-4.460053	2.584601	4.608812
H	-4.457764	4.590845	0.534722
H	-4.656740	3.029065	-1.434623
H	-5.740117	-0.197877	1.275968
H	-5.544544	1.318142	3.200152
H	-6.241610	-1.271781	-0.679202
H	-4.480279	-1.209936	-0.341531
H	-5.891968	-1.532409	-3.087136
H	-4.950779	-2.775742	-2.212288
C	-7.098753	-3.172923	2.583802
C	-6.603477	-3.877187	1.464888
C	-5.244494	-3.738765	1.082535
C	-4.394851	-2.861594	1.800324
C	-4.897360	-2.131267	2.907572
C	-6.248549	-2.306325	3.303228
C	-4.777755	-4.513258	-0.125317
O	-5.590644	-4.761595	-1.094013
N	-3.480020	-4.946283	-0.109237
C	-2.931635	-5.818915	-1.161130
C	-1.642047	-5.294747	-1.796425
O	-1.986437	-4.439159	-2.965783
C	-0.885239	-4.010785	-3.733157

C	0.452329	-4.030917	-3.268300
C	1.504993	-3.608420	-4.113416
C	1.219468	-3.151498	-5.419741
C	-0.121500	-3.130250	-5.885939
C	-1.168057	-3.556076	-5.047455
O	2.194615	-2.681997	-6.326354
C	3.609100	-2.857025	-5.926981
C	4.482676	-2.251509	-7.009803
O	4.535510	-0.793089	-6.806301
C	5.300782	-0.113409	-7.865019
C	5.508538	1.334684	-7.458599
O	4.214388	2.038888	-7.504995
C	4.352090	3.445187	-7.091924
C	2.969951	4.093919	-7.041099
O	2.041142	3.494960	-6.066831
C	2.572617	3.480199	-4.693687
C	1.373007	3.231602	-3.781189
O	1.941095	2.709259	-2.525360
C	1.038870	2.314504	-1.519947
C	-0.373197	2.379947	-1.642909
C	-1.184223	1.980287	-0.561253
C	-0.598518	1.521197	0.642154
C	0.813997	1.435085	0.754585
C	1.626079	1.831528	-0.327606
O	-1.490177	1.190935	1.674717
C	-0.895342	0.658770	2.919519
C	-2.043595	0.259446	3.836017

N	-2.821300	-0.841113	3.242305
C	-4.094212	-1.100429	3.665754
O	-4.620515	-0.458018	4.651411
H	-8.143700	-3.292982	2.888151
H	-7.253741	-4.537428	0.882042
H	-3.361283	-2.727877	1.464236
H	-6.615588	-1.734926	4.161593
H	-2.704538	-6.804445	-0.709893
H	-3.706768	-5.969270	-1.931105
H	-1.038812	-4.721536	-1.069221
H	-1.036022	-6.146017	-2.165572
H	0.692137	-4.355374	-2.252765
H	2.528023	-3.641697	-3.731222
H	-0.330204	-2.787406	-6.904135
H	-2.203466	-3.548756	-5.401749
H	3.807559	-2.354471	-4.961001
H	3.822753	-3.938311	-5.821071
H	5.507971	-2.670646	-6.931207
H	4.078207	-2.491112	-8.014696
H	6.293795	-0.597119	-7.983034
H	4.757936	-0.180137	-8.830676
H	6.221012	1.812725	-8.164006
H	5.931399	1.390095	-6.433856
H	4.974548	3.998462	-7.828369
H	4.861419	3.497411	-6.107458
H	3.106618	5.173627	-6.815133
H	2.459088	3.993381	-8.013625

H	3.308239	2.662798	-4.565584
H	3.065531	4.445001	-4.456160
H	0.803236	4.159692	-3.584512
H	0.693708	2.490696	-4.240771
H	-0.847075	2.741879	-2.559211
H	-2.274057	2.024979	-0.638144
H	1.289448	1.079755	1.671202
H	2.716507	1.780285	-0.251759
H	-0.278690	1.438212	3.404427
H	-0.264654	-0.214333	2.672378
H	-2.714751	1.116117	4.023383
H	-1.618375	-0.038275	4.815557
H	-2.865360	-4.797890	0.727709
H	-2.350293	-1.377774	2.483170
S	-0.456384	-3.595045	1.638523
O	-1.631098	-4.736575	1.953209
O	-1.155217	-2.095655	1.392830
O	0.571596	-4.047392	0.421757
C	0.591733	-3.416023	3.189595
C	-0.013602	-2.904820	4.352844
C	1.941737	-3.794120	3.129924
H	-1.072659	-2.628688	4.373355
H	2.367465	-4.191495	2.203542
C	0.790642	-2.752907	5.502031
C	2.728026	-3.630454	4.293752
H	0.343248	-2.352837	6.418386
H	3.787264	-3.908427	4.268156

C	2.169791	-3.102942	5.487682
C	3.030970	-2.866798	6.711591
H	2.494948	-3.125147	7.642253
H	3.966401	-3.450715	6.671426
H	3.308027	-1.796391	6.783537
Cl	-1.907669	-1.573585	-1.214442
Cl	2.163035	-2.053416	-0.710417

Table S16: Cartesian Coordenates of **4**.TS$^-$

**4**.TS$^-$	x	y	z
N	1.204870	0.203951	-6.009969
C	2.230742	0.115087	-5.113107
C	1.963114	-0.162572	-3.757403
C	0.624661	-0.355592	-3.333612
C	-0.417133	-0.268552	-4.287878
C	-0.092537	0.017081	-5.629633
C	1.520277	0.412160	-7.451805
C	3.108995	-0.262453	-2.859757
N	4.355060	0.276544	-3.196345
N	5.254607	-0.002354	-2.224021
N	4.566928	-0.743300	-1.236614
C	3.259655	-0.891462	-1.615658
C	5.295207	-1.230487	-0.051680
C	5.232753	-0.260288	1.126332
O	3.892895	-0.405299	1.720394
C	3.641570	0.301023	2.917143
C	4.468131	1.325001	3.432473

C	4.102844	1.984497	4.633124
C	2.917879	1.611602	5.312763
C	2.098345	0.576406	4.791271
C	2.459011	-0.073930	3.600814
O	2.467679	2.193491	6.510442
C	3.178279	3.398513	6.987896
C	2.429298	3.911279	8.215972
O	1.066123	4.382858	7.930085
C	1.022564	5.706738	7.293417
C	-0.395197	6.026055	6.890923
C	-1.445191	5.173782	7.013720
C	-2.857452	5.520712	6.627382
O	-3.319611	4.508865	5.644387
C	-4.786793	4.511215	5.503224
C	-5.166950	3.420368	4.520168
O	-4.927739	3.928119	3.148342
C	-5.050854	2.992237	2.103714
C	-4.818194	3.484669	0.791864
C	-4.863591	2.609206	-0.310174
C	-5.138472	1.233060	-0.107262
C	-5.405184	0.748507	1.192051
C	-5.367428	1.627230	2.298514
O	-5.125136	0.409754	-1.257555
C	-5.028813	-1.039484	-0.994376
C	-4.884224	-1.742040	-2.341375
N	-3.769583	-1.207878	-3.141330
N	-4.010292	-0.757146	-4.455622

N	-2.817231	-0.349881	-4.950095
C	-1.820681	-0.523343	-3.984251
C	-2.445022	-1.058587	-2.848437
H	3.242673	0.204436	-5.512958
H	0.400991	-0.586759	-2.290530
H	-0.856358	0.081902	-6.404569
H	1.578842	-0.576089	-7.936208
H	0.718140	1.010000	-7.908618
H	2.484079	0.936959	-7.530807
H	6.343418	-1.359517	-0.364302
H	4.884817	-2.211732	0.236883
H	5.407136	0.779425	0.791525
H	6.005263	-0.539200	1.868183
H	5.386382	1.627511	2.921314
H	4.751320	2.775715	5.019411
H	1.186660	0.293216	5.325421
H	1.836418	-0.870485	3.187344
H	4.216056	3.136132	7.277442
H	3.215304	4.153727	6.178791
H	3.032181	4.717437	8.685358
H	2.301954	3.092015	8.944056
H	1.409959	6.476792	7.998434
H	1.674789	5.736062	6.392953
H	-0.542133	7.029796	6.465131
H	-1.285632	4.173492	7.434264
H	-3.524222	5.480266	7.517893
H	-2.924960	6.537573	6.187129

H	-5.149599	5.500359	5.153086
H	-5.256557	4.292736	6.486482
H	-6.237679	3.157697	4.627307
H	-4.553318	2.518509	4.707493
H	-4.586236	4.545045	0.650168
H	-4.672485	2.977422	-1.323244
H	-5.635982	-0.304187	1.367241
H	-5.560903	1.222433	3.294554
H	-5.948044	-1.415826	-0.510438
H	-4.168563	-1.232159	-0.329425
H	-5.784956	-1.600311	-2.958249
H	-4.735093	-2.821553	-2.175042
C	-7.006431	-3.354557	3.067873
C	-6.617518	-3.990714	1.869241
C	-5.335209	-3.742364	1.315069
C	-4.455951	-2.831210	1.950867
C	-4.858357	-2.164107	3.136466
C	-6.131755	-2.442961	3.696423
C	-4.994012	-4.432468	0.017132
O	-5.918480	-4.708626	-0.836069
N	-3.676049	-4.751797	-0.183798
C	-3.254736	-5.572949	-1.334519
C	-1.897644	-5.165178	-1.907874
O	-2.111681	-4.153214	-2.986532
C	-0.954368	-3.829203	-3.731694
C	0.350936	-3.825375	-3.182535
C	1.458642	-3.494634	-3.998641

C	1.256237	-3.140909	-5.353419
C	-0.053940	-3.139198	-5.898948
C	-1.153842	-3.487617	-5.092789
O	2.283716	-2.749483	-6.237086
C	3.676646	-2.908790	-5.761798
C	4.599792	-2.362607	-6.835550
O	4.627351	-0.894247	-6.718437
C	5.424402	-0.259741	-7.781261
C	5.632166	1.200529	-7.419028
O	4.340307	1.907197	-7.496016
C	4.478576	3.322011	-7.111594
C	3.099672	3.979448	-7.092297
O	2.157445	3.414912	-6.110287
C	2.666885	3.455804	-4.728897
C	1.453980	3.235199	-3.825657
O	1.999246	2.718008	-2.556690
C	1.080243	2.318484	-1.568266
C	-0.329814	2.370761	-1.717513
C	-1.158617	1.964314	-0.650758
C	-0.591117	1.514935	0.565833
C	0.820201	1.435831	0.701150
C	1.648889	1.838327	-0.365448
O	-1.494622	1.198363	1.591437
C	-0.910487	0.724831	2.865602
C	-2.063039	0.376646	3.795945
N	-2.824574	-0.771015	3.275289
C	-4.040530	-1.087377	3.810439

O	-4.508073	-0.456551	4.833437
H	-7.990311	-3.559273	3.502711
H	-7.293830	-4.679103	1.352440
H	-3.483058	-2.620497	1.494538
H	-6.423027	-1.914788	4.609790
H	-3.182148	-6.630751	-1.009923
H	-4.035582	-5.521368	-2.111440
H	-1.234914	-4.752523	-1.126382
H	-1.403216	-6.043616	-2.365822
H	0.513729	-4.052477	-2.123489
H	2.458699	-3.506152	-3.557487
H	-0.196599	-2.875755	-6.951616
H	-2.166885	-3.500487	-5.506608
H	3.833140	-2.355208	-4.817241
H	3.878725	-3.983544	-5.588664
H	5.623212	-2.764169	-6.679414
H	4.248794	-2.668294	-7.842516
H	6.417554	-0.751635	-7.856351
H	4.906313	-0.358624	-8.757611
H	6.351386	1.655143	-8.133013
H	6.046708	1.286156	-6.393012
H	5.112696	3.855992	-7.852306
H	4.976389	3.392999	-6.122415
H	3.240730	5.064437	-6.896451
H	2.597504	3.854170	-8.066506
H	3.403662	2.647107	-4.558080
H	3.153028	4.431132	-4.522856

H	0.893760	4.171837	-3.643559
H	0.770603	2.498834	-4.285910
H	-0.789350	2.731104	-2.641630
H	-2.247540	2.005502	-0.746748
H	1.278007	1.083736	1.627463
H	2.738114	1.797897	-0.267752
H	-0.293808	1.525995	3.313754
H	-0.282876	-0.161590	2.665415
H	-2.740610	1.240861	3.917679
H	-1.646847	0.151298	4.797930
H	-2.970930	-4.645983	0.589305
H	-2.391234	-1.289413	2.480035
S	-0.538185	-3.421218	1.393632
O	-1.663332	-4.617254	1.697770
O	-1.286553	-1.934161	1.285079
O	0.435588	-3.767956	0.102518
C	0.581933	-3.332981	2.903005
C	0.025180	-2.921154	4.128808
C	1.930686	-3.689567	2.755611
H	-1.033805	-2.657373	4.215365
H	2.316977	-4.006478	1.782050
C	0.877056	-2.853793	5.251300
C	2.765798	-3.612887	3.894664
H	0.467470	-2.531633	6.214948
H	3.824754	-3.877629	3.802036
C	2.256131	-3.191250	5.150607
C	3.164486	-3.062446	6.356902

H	2.704790	-3.504897	7.259240
H	4.138657	-3.551142	6.185713
H	3.357809	-1.995712	6.583841
F	-1.925473	-1.415219	-1.632136
F	2.364470	-1.603725	-0.860196

Table S17: Cartesian Coordenates of **5**.TS^{-}

**5**.TS^{-}	x	y	z
N	1.131752	0.084208	-6.172379
C	2.142197	-0.083452	-5.269933
C	1.856537	-0.163692	-3.891566
C	0.508587	-0.113940	-3.463043
C	-0.528407	0.065067	-4.417926
C	-0.175786	0.168828	-5.778420
C	1.470260	0.123364	-7.623484
C	2.995096	-0.220784	-2.973900
N	4.162025	0.493041	-3.279700
N	5.060304	0.309297	-2.290388
N	4.440512	-0.537491	-1.342273
C	3.160639	-0.895198	-1.737791
C	5.208330	-0.861752	-0.127303
C	5.067757	0.182791	0.980909
O	3.739740	-0.000567	1.595135
C	3.487063	0.690304	2.798194
C	4.330726	1.686095	3.341437
C	3.995749	2.298499	4.575310
C	2.819402	1.909882	5.260163

C	1.962418	0.929560	4.693742
C	2.295048	0.321252	3.473022
O	2.418148	2.413247	6.511139
C	3.219522	3.512709	7.087055
C	2.621257	3.850742	8.451319
O	1.232119	4.325733	8.400978
C	1.088189	5.708476	7.925156
C	-0.372213	6.002297	7.687529
C	-1.368055	5.087455	7.809956
C	-2.820982	5.380614	7.553275
O	-3.266835	4.458631	6.478694
C	-4.732357	4.434988	6.339064
C	-5.073345	3.511270	5.184264
O	-4.761783	4.228601	3.928960
C	-4.877379	3.499715	2.730327
C	-4.536451	4.207137	1.546568
C	-4.600362	3.564329	0.297615
C	-5.003383	2.206593	0.220756
C	-5.355055	1.503585	1.393922
C	-5.291475	2.149276	2.651626
O	-5.011045	1.641818	-1.074109
C	-5.332694	0.206238	-1.149630
C	-5.291328	-0.197770	-2.620131
N	-4.003972	0.060527	-3.294908
N	-4.056588	0.831755	-4.480343
N	-2.794311	0.899292	-4.946227
C	-1.948882	0.187541	-4.084086

C	-2.712141	-0.355036	-3.015791
H	3.152685	-0.192578	-5.670811
H	0.275769	-0.141296	-2.398390
H	-0.923795	0.313415	-6.558171
H	1.445489	-0.904662	-8.020964
H	0.729731	0.743492	-8.148926
H	2.475833	0.556764	-7.733622
H	6.268384	-0.917073	-0.424280
H	4.896055	-1.846710	0.247623
H	5.173402	1.209169	0.581405
H	5.856884	0.003803	1.736898
H	5.245124	1.996992	2.828537
H	4.664077	3.061203	4.984215
H	1.054270	0.639088	5.230127
H	1.654008	-0.453143	3.042742
H	4.268694	3.184000	7.232734
H	3.211695	4.377767	6.395740
H	3.276598	4.600024	8.944452
H	2.585702	2.942375	9.076722
H	1.515320	6.412192	8.674671
H	1.648953	5.864257	6.977636
H	-0.597753	7.034333	7.380799
H	-1.123897	4.061114	8.109166
H	-3.429928	5.188902	8.465153
H	-2.980390	6.434051	7.240967
H	-5.127924	5.455497	6.152024
H	-5.196048	4.043413	7.270200

H	-6.149695	3.249924	5.202790
H	-4.474360	2.582794	5.252914
H	-4.218078	5.251892	1.620475
H	-4.332233	4.098628	-0.619369
H	-5.662547	0.455897	1.362160
H	-5.543621	1.567025	3.541133
H	-6.356889	0.017897	-0.773623
H	-4.613672	-0.368543	-0.536366
H	-6.041689	0.370443	-3.192402
H	-5.538059	-1.270184	-2.694484
C	-7.166274	-3.976309	2.115058
C	-6.526416	-4.899800	1.261056
C	-5.143296	-4.767284	0.972816
C	-4.410543	-3.688144	1.530946
C	-5.052006	-2.751843	2.383855
C	-6.432140	-2.910687	2.676674
C	-4.544018	-5.781282	0.022522
O	-5.295603	-6.387754	-0.826271
N	-3.195484	-6.020754	0.121284
C	-2.524929	-7.016693	-0.733279
C	-1.237529	-6.512573	-1.389830
O	-1.607723	-5.505168	-2.412666
C	-0.564300	-4.946142	-3.173810
C	0.800516	-5.291046	-3.039773
C	1.773186	-4.678543	-3.868393
C	1.380841	-3.720800	-4.831940
C	0.010572	-3.378987	-4.967323

C	-0.953906	-3.983663	-4.143846
O	2.267745	-3.061912	-5.712985
C	3.686748	-3.470985	-5.640273
C	4.449019	-2.802441	-6.768186
O	4.644065	-1.375521	-6.460491
C	5.499816	-0.719928	-7.466343
C	5.663444	0.742237	-7.094415
O	4.391376	1.447702	-7.338853
C	4.532090	2.893674	-7.095552
C	3.173914	3.576953	-7.246836
O	2.160092	3.175710	-6.256654
C	2.607588	3.381719	-4.866461
C	1.350783	3.335461	-3.997291
O	1.832749	2.993211	-2.643603
C	0.894539	2.512110	-1.712332
C	-0.512096	2.597814	-1.874717
C	-1.362757	2.074633	-0.878212
C	-0.817590	1.476222	0.284454
C	0.590648	1.383698	0.438967
C	1.439923	1.905397	-0.557221
O	-1.741193	1.013393	1.235725
C	-1.186804	0.321557	2.419543
C	-2.358734	-0.145882	3.272580
N	-3.079407	-1.274215	2.654158
C	-4.359742	-1.572928	3.035335
O	-4.977716	-0.873375	3.923230
H	-8.231836	-4.087011	2.341749

H	-7.081464	-5.726410	0.806437
H	-3.353498	-3.568868	1.275502
H	-6.906900	-2.185201	3.344633
H	-2.252641	-7.902907	-0.125057
H	-3.244838	-7.344014	-1.501743
H	-0.544049	-6.051000	-0.661165
H	-0.733185	-7.368315	-1.881474
H	1.126331	-6.020741	-2.294741
H	2.822092	-4.955568	-3.736395
H	-0.287347	-2.649605	-5.725641
H	-2.012142	-3.727706	-4.247478
H	4.112438	-3.190285	-4.657610
H	3.759351	-4.569610	-5.764625
H	5.439779	-3.296742	-6.860639
H	3.907594	-2.919018	-7.729761
H	6.497936	-1.207306	-7.477475
H	5.048908	-0.817045	-8.476023
H	6.463891	1.185713	-7.723915
H	5.953130	0.844538	-6.027836
H	5.230310	3.332442	-7.841366
H	4.960438	3.064324	-6.086614
H	3.339015	4.674940	-7.188846
H	2.725868	3.335328	-8.225677
H	3.293011	2.571943	-4.551436
H	3.130187	4.355764	-4.771710
H	0.816919	4.304112	-3.979300
H	0.665195	2.554643	-4.371243

H	-0.951586	3.057931	-2.764010
H	-2.448827	2.121971	-0.995651
H	1.035135	0.919183	1.320227
H	2.526180	1.834295	-0.451015
H	-0.566113	1.027841	3.002369
H	-0.559771	-0.528140	2.087770
H	-3.066856	0.682679	3.439949
H	-1.962382	-0.440560	4.265417
H	-2.619619	-5.611917	0.891053
H	-2.538727	-1.862287	1.987374
S	-0.425059	-3.970788	1.568682
O	-1.416770	-5.198218	2.083787
O	-1.331805	-2.675116	0.982320
O	0.700378	-4.462134	0.449307
C	0.537385	-3.362902	3.062365
C	-0.179401	-2.870745	4.166373
C	1.937643	-3.471327	3.035479
H	-1.272464	-2.815446	4.162706
H	2.451298	-3.875843	2.159204
C	0.559646	-2.459376	5.298050
C	2.651741	-3.055685	4.179637
H	0.024595	-2.072855	6.171910
H	3.744780	-3.127790	4.182356
C	1.978190	-2.546713	5.322967
C	2.763257	-2.072063	6.527849
H	2.123108	-1.983404	7.421959
H	3.600188	-2.754639	6.759916

H	3.201378	-1.074258	6.331026
Te	-2.058667	-1.531351	-1.363165
Te	1.850549	-2.280144	-0.771974
C	-3.582990	-3.032711	-1.728377
H	-3.803085	-3.045204	-2.807142
H	-3.145423	-3.998835	-1.442339
H	-4.488897	-2.813556	-1.146585
C	3.389812	-3.819340	-0.693426
H	2.875103	-4.776254	-0.865886
H	4.120194	-3.630114	-1.495845
H	3.887384	-3.835062	0.288631
